# Optimal Stimulation Sites and Connectomes for GPi and STN‐DBS in Cervical Dystonia

**DOI:** 10.1111/cns.70561

**Published:** 2025-08-17

**Authors:** Tao Xue, Youjia Qiu, Wei Tian, Hutao Xie, Shiying Fan, Houyou Fan, Minjia Xie, Ming Ye, Zhong Wang, Tongbo Ning, Chunlei Han, Hua Zhang, Anchao Yang, Lin Sang, Jurgen Germann, Alexandre Boutet, Joseph Tam, Andres M. Lozano, Fangang Meng, Yutong Bai, Jianguo Zhang

**Affiliations:** ^1^ Department of Neurosurgery, Beijing Tiantan Hospital Capital Medical University Beijing China; ^2^ Department of Neurosurgery & Brain and Nerve Research Laboratory The First Affiliated Hospital of Soochow University Suzhou China; ^3^ Department of Neurosurgery Weihai Central Hospital Weihai China; ^4^ Department of Pediatric Surgery Children's Hospital of Capital Institute of Pediatric Beijing China; ^5^ Department of Neurosurgery Beijing FengTai Hospital Beijing China; ^6^ Krembil Research Institute University Health Network, University of Toronto Toronto Ontario Canada; ^7^ Joint Department of Medical Imaging University of Toronto Toronto Ontario Canada; ^8^ Division of Neurosurgery, Depatment of Surgery University of Toronto Toronto Canada; ^9^ Beijing Neurosurgical Institute Capital Medical University Beijing China

## Abstract

**Aims:**

To map optimal stimulation targets (sweet spots) and neural networks for globus pallidus internus (GPi)‐ and subthalamic nucleus (STN)‐deep brain stimulation (DBS) in cervical dystonia (CD), and compare their structural/functional connectivity profiles and predictive validity for clinical outcomes.

**Methods:**

Retrospective analysis of 76 stimulation settings from 38 CD patients across four centers. Volume of tissue activated was reconstructed; connectivity‐based sweet spots were identified. Structural/functional connectivity models were developed using normative connectomes and validated externally. Clinical outcomes were assessed using validated scales.

**Results:**

Optimal targets localized to the posterior ventral medial GPi and dorsolateral STN. The ideal probabilistic stimulation maps of STN‐DBS exhibited predictive clinical improvement. Both targets showed beneficial connections to the motor cortex, with GPi‐DBS negatively connected to the occipital lobe and STN‐DBS positively connected to the premotor cortex and cerebellum. Functional connectivity patterns further highlighted shared and distinct regions linked to CD symptoms. Moreover, the structural and functional connectivity models predicted postoperative improvement through internal and external validation.

**Conclusion:**

GPi‐ and STN‐DBS engage distinct but overlapping networks in CD. Connectivity‐based models robustly predict clinical improvement, offering tools for personalized targeting and programming. These findings clarify network mechanisms of DBS in dystonia and advance precision neuromodulation strategies.

## Graphical abstract

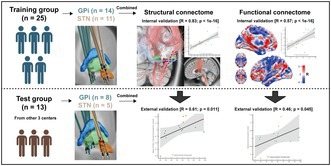

## 1 Introduction

Cervical dystonia (CD) is the most common type of focal dystonia, with an estimated prevalence of 1.18 per 100,000 persons year [1]. It typically affects patients aged approximately 40 years and is slightly more common in females [2]. CD is characterized by inappropriate contractions of neck muscles associated with dysfunctions of sensorimotor networks, resulting in involuntary movements of the head, neck, and shoulder, accompanied by tremors and sustained abnormal postures [3]. Patients with CD generally experience pain, disability, and stigma, leading to a decreased quality of life [4]. Although botulinum toxin is acknowledged as the first line of treatment for CD, approximately one‐third of patients fail to respond to botulinum neurotoxin injections, and 20% of patients may discontinue injection due to lack of long‐term efficacy and side effects [5]. Patients with persistent symptoms may require other interventions.

Although current observations support the important role of basal ganglia in CD, the refined evidence is still limited. Recent evidence even attributes the pathophysiology of CD to the miscommunication between basal ganglia and cerebellar loop [6]. As indispensable components of basal ganglia based on physiology and functionality, the globus pallidus internus (GPi) and subthalamic nucleus (STN) are interrelated and different from each other. Deep brain stimulation (DBS) is a well‐established treatment for pharmaco‐resistant CD through a procedure with multiple mechanisms, such as long‐term neuronal reorganization, intermittent neural modulation, and synaptic plasticity [7]. The GPi is the primary target for CD, whereas the STN is an alternative and promising target for treating CD [8]. Both are crucial hubs of the motor circuit, and the clinical benefits from them could be attributed to positive or negative modulations of fiber pathway activity [9]. A previous meta‐analysis found that the two targets have similar efficacies [10]. Moreover, several studies concluded that the effectiveness of DBS may depend on the modulation of functional brain regions connected to the stimulation site, and that connectivity between brain regions and stimulation sites was a crucial factor in the DBS response [11, 12, 13]. Although it is believed that DBS relieves CD by stimulating local regions and normalizing the pathological networks, it remains unclear which specific sites, fiber tracts, and functional networks lead to these effects [14]. Reich et al. firstly defined sweet and sour spots to quantify statistically significant clusters and visualize the volumes of high and low likelihood of good outcomes [15]. Considering the different spatial locations of implanted electrodes may exert similarity in cortical volumes for optimal clinical benefit, it is meaningful to identify the optimal stimulation site within statistical thresholding for DBS [15]. Horn et al. have compared the optimal stimulation site of GPi‐DBS between generalized dystonia and CD, and they found that modulation of the striatopallidofugal axis of the basal ganglia accounted for the optimal treatment of CD [16]. However, there is a need for studies comparing the refined stimulation sites of GPi and STN‐DBS in CD.

We thus evaluated the relationships between electrode location and stimulation‐dependent connectivity profiles on the clinical improvement of CD patients following GPi‐DBS and STN‐DBS. First, we identified neural substrates locally stimulated after GPi‐DBS and STN‐DBS implantation in patients with CD; then, using state‐of‐the‐art normative data, we determined the optimal structural and functional networks associated with clinical outcomes and examined specificities and discrepancies in the optimal networks between the GPi‐DBS and STN‐DBS groups [16, 17].

## 2 Methods

### 2.1 Patients and Neuroimaging

We conducted a multicenter retrospective study that enrolled 38 CD patients who underwent GPi‐DBS (*n* = 22) or STN‐DBS (*n* = 16) surgery from 2017 to 2022 at four centers in China. This study was approved by the local Institutional Review Boards (KY2022‐006‐02), and all participants provided written informed consent. The datasets were as follows: 25 patients (GPi/STN: 14/11) from Beijing Tian Hospital used as training data and 13 patients (8/5) from three other centers (six for Beijing Fengtai Hospital, five for the First Affiliated Hospital of Soochow University and two for Weihai Central Hospital) used as unseen validation data. All patients received preoperative evaluation involving a neurological examination, MRI, and neuropsychological testing to exclude severe or structural psychological comorbidities. Detailed inclusion and exclusion criteria can be found in Table S1. The improvement of CD was assessed by two movement disorder neurologists blinded to stimulation conditions using the Toronto Western Spasmodic Torticollis Rating Scale (TWSTRS) based on a standard video recording [18]. The senior author (JGZ) made the final decision of any disputed assessment (difference > 1 point). The improvement in total TWSTRS scores was presented as ΔTWSTRS, calculated as (TWSTRS_pre_ − TWSTRS_post_)/TWSTRS_pre_. The Shapiro–Wilk test was used to assess the normality of continuous variables. Parametric tests were used for normally distributed data, and non‐parametric tests were applied otherwise. Categorical variables were compared using Fisher's exact test. Since multiple subscales of the TWSTRS were used, Bonferroni correction was used for multiple comparisons, with a *p*_Bonferroni_ value < 0.05 being statistically significant. In addition, non‐motor symptoms were also assessed pre‐ and post‐operation. The Montreal Cognitive Assessment (MoCA), Hamilton Rating Scale for Anxiety (HAMA), and Hamilton Rating Scale for Depression (HRSD) were used to evaluate the severities of cognitive function, anxiety, and depression, respectively. Percentage change was used to assess the change on these scales after surgery. Moreover, the age, sex, preoperative scales, follow‐up durations, and programming parameters were recorded and tested for their predictions of clinical outcome by using univariate analysis (Table S2) [19].

### 2.2 Surgical Procedure

A 3T magnetic resonance image (MRI) with high resolution (1 × 1 × 1 mm^3^) of every patient's head was obtained 1 day before the surgery. Then, a high spatial resolution computed tomography (CT) head scan (spacing 0.625 mm) was conducted with a Leksell stereotactic frame mounted on the head on the day of the surgical procedure. The CT and MRI images were coregistered to determine the implantation trajectory plan and localize the DBS contacts. DBS electrode implantation was performed under local anesthesia, using a Leksell microstereotactic system (Elekta Instrument AB, Stockholm, Sweden).

Intraoperative microelectrode recording measuring the length of the DBS trajectory in the two targets and macro‐stimulation tests were used for trajectory selection. The tip of the microelectrode was guided to the dorsolateral portions of the STN based on the implantation trajectory plan. Microelectrode recording started 15 mm above the predefined target, and the recorded neuronal activity was used to help define the boundary of GPi/STN during surgery. The side‐specific effects of intraoperative stimulations were also used to confirm the optimal location. If intraoperative stimulation induced adverse reactions in patients, the position of the electrode would be readjusted until satisfactory effects were achieved.

After the target location was confirmed, the microelectrode was pulled out and a quadripolar electrode (PINS‐L301 or Medtronic‐3389 for STN and PINS‐L302 or Medtronic‐3387 for GPi) was implanted along the microelectrode trajectory to the position of the two targets. Next, post‐operative high spatial resolution CT (spacing 0.625 mm) was performed to exclude intracranial hemorrhage and to verify the exact location of the electrodes by merging them with the preoperative MR images. The second‐stage operation was performed after testing without obvious side effects during the plug‐in period; the electrodes were then connected to an implantable pulse generator (IPG) implanted in the subclavicular area under general anesthesia. About 1 month after the surgery, DBS is switched on, and the programming parameters are adjusted by professional programming doctors to ensure that the efficacy is maximized and the side effects are minimized. The detailed information on the stereotactic coordinates is shown in Table S3.

### 2.3 Lead Localization and Volume of Tissue Activated Estimation

The processing pipeline in the Lead‐DBS v2.5 toolbox was used to localize DBS electrodes (www.lead‐dbs.org) [20]. Briefly, postoperative CT and preoperative MRI were co‐registered using linear transform through advanced normalization tools (ANTs) (Figure S1A) [21]. A subcortical refinement step was added for brain shift correction which was caused by acute intra‐cranial postoperative changes. Then, images were normalized into the MNI space (2009b, asymmetric, non‐linear) using the symmetric diffeomorphic registration (SyN) method implemented in ANTs with the preset “effective: low variance default + subcortical refinement” (Figure S1B) [21, 22]. DBS leads were automatically pre‐reconstructed using PaCER approach and manually refined when necessary [23]. Estimation of volumes of tissue activated (VTA) was described previously (Figure S1C) [19]. Briefly, the electric fields were estimated using a finite element method on a four‐compartment mesh that includes gray matter, white matter, electrode contacts, and insulated sections (Figure S1C). Subcortical gray matter nuclei were defined by the DISTAL atlas [24]. The VTA was evaluated with the Lead‐DBS pipeline based on finite element models (FEM). Patient‐specific stimulation parameters, especially activated contacts, pulse width, frequency and amplitudes, were used to calculate VTAs using the SimBio/FieldTrip pipeline [25]. The dispersion of the electric field throughout the tissue was estimated using a homogeneous conductivity of = 0.1 S/m. The binary VTA threshold was set to *e* = 0.2 V/mm [26].

### 2.4 Probabilistic Stimulation Maps (PSMs) and Voxel‐Wise Statistical Analysis

A PSM was generated to identify stimulation sites associated with clinical improvement in cervical dystonia, as measured by the TWSTRS score. First, VTAs were constructed for each active contact and non‐linearly normalized to MNI space using Lead‐DBS. To allow for group‐level analysis, VTAs from the left hemisphere were flipped to the right hemisphere. Next, each voxel was included in the analysis only if it was covered by at least 20% of all VTAs, ensuring statistical robustness. Each patient's VTA was then weighted by the percentage change in TWSTRS score, and the average weighted improvement was calculated for each voxel, producing a voxel‐wise mean effect map. To evaluate statistical significance, a two‐sample t‐test was performed voxel‐wise, comparing clinical outcomes between VTAs that included the voxel and those that did not. Voxels showing significant changes (*p* < 0.05) were retained to create the final PSM. Voxels with significantly negative mean values (i.e., improvement in TWSTRS scores) were labeled as “sweet spots”, whereas those with significantly positive mean values (i.e., worsening symptoms) were considered “sour spots” (Figure S1D). Patients whose VTAs showed greater overlap with sweet spots and less overlap with sour spots were considered more likely to experience clinical benefit.

Finally, to validate the predictive reliability of the PSM, leave‐one‐out cross‐validation (LOOCV) was applied. For each iteration, the PSM was recalculated using all patients except one, and the model was tested on the excluded patient by computing their PSM score, which was defined as the sum of the weighted voxel values within the overlap between their VTA and the PSM. The predicted score was then compared to the actual percentage change in TWSTRS score, which served as the clinical label. The correlation between predicted and observed values across all iterations was assessed using Spearman correlation.

### 2.5 Connectivity Estimation

Structural connectivity analysis (Figure S1E) was performed using a normative diffusion MRI connectome from the Human Connectome Project (HCP), implemented via Lead‐Connectome [27, 28]. White matter fiber tracts traversing each patient's VTA were extracted, and a total of 20,000 streamlines per subject were sampled using generalized q‐sampling in DSI Studio. For each fiber, a fiber *T*‐score was assigned by conducting two‐sample t‐tests comparing the percentage change in TWSTRS scores between connected and disconnected VTAs [29]. These *T*‐scores were used to quantify the association between fiber modulation and clinical outcomes [30].

Functional connectivity analysis (Figure S1F) was conducted using a normative resting‐state fMRI connectome derived from the Brain Genomics Superstruct Project [31, 32]. For each patient, the VTA was used as a seed region, and voxel‐wise correlations with the rest of the brain were calculated across the normative dataset [19]. These correlations were averaged, Fisher Z‐transformed, and used to construct a patient‐specific functional connectivity fingerprint. The voxel‐wise correlation between each patient's connectivity fingerprint and their TWSTRS improvement score was computed across the cohort, generating a group‐level R‐map that reflects the optimal connectivity profile associated with clinical benefit [23, 33].

To assess the predictive value of the connectivity‐based models, a leave‐one‐patient‐out cross‐validation (LOOCV) was performed, consistent with the method described above [23]. This approach was applied to both structural and functional connectivity models, and the predicted scores were correlated with the actual TWSTRS improvement. Additionally, a combined model across both STN and GPi‐DBS cohorts was constructed to evaluate the overall network predictive value. To compare target‐specific functional connectivity profiles, an agreement map was generated by identifying voxels with consistent directional effects in both R‐maps [34]. The impact of clinical and demographic covariates (such as age at surgery, sex, etc.) on model performance was further examined using general linear models.

### 2.6 Internal and External Validation

Internal validation of the above‐mentioned models was performed by the LOOCV method to assess whether the PSM/fibers/network‐based model can predict TWSTRS scores (Figure S1G) [23]. Three of eight internally validated models that showed good predictive results (*p*_*i*_ < 0.05) were externally validated by inputting test group data to explore the robustness and generalizability of the model (Figure S1H).

### 2.7 Additional Analysis

We analyzed the influence of covariates (sex, age at surgery, preoperative HAMA, HRSD, and MoCA) on the PSMs and structural/functional connectivity models; we conducted a sensitivity analysis using ΔTWSTRS_severity_.

## 3 Results

### 3.1 Clinical Data

This study includes 38 patients diagnosed with CD who received GPi‐DBS (*n* = 22) or STN‐DBS (*n* = 16) implantation. In the training group, a significant sex difference was observed between the two targets (*p* = 0.0027), and the GPi‐DBS group had longer follow‐ups than the STN‐DBS group (*p* = 0.0057). Patients with CD had a significant improvement in total TWSTRS score (*p* < 0.0001), HAMA score (*p* = 0.0001), and HRSD score (*p* = 0.0018) at the 37.29 ± 15.02 months follow‐ups. A total of 76 electrodes were implanted, and the stimulation parameters were similar except for voltage (*p* < 0.0001). Both targets had improvement in TWSTRS total (GPi: *p* < 0.0001, STN: *p* < 0.0001), severity (GPi: *p* < 0.0001, STN: *p* < 0.0001), disability (GPi: *p* = 0.0002, STN: *p* = 0.004), and pain (GPi: *p* = 0.002, STN: *p* = 0.0078) scores at follow‐ups. For non‐motor symptoms, both target groups showed improvement in the HAMA score (GPi: *p* = 0.0266, STN: *p* < 0.0001), whereas only the STN‐DBS group showed improvement in the HRSD score (*p* < 0.0001). However, no statistical difference was found in the MoCA score. No statistical difference was observed between the two targets in improving total TWSTRS, severity, disability, pain scores, and non‐motor symptom scales. In addition, the GPi‐DBS group showed significantly higher voltages than the STN‐DBS group (*p* < 0.0001). Furthermore, univariate regression analysis of the improvements of TWSTRS total scores indicated that preoperative age at surgery (*p* = 0.0017), HAMA score (*p* = 0.0017), HRSD score (*p* = 0.0228), and MoCA score (*p* = 0.0053) might be predictive markers for the improvement of TWSTRS in CD patients (Table S2).

**FIGURE 1:**
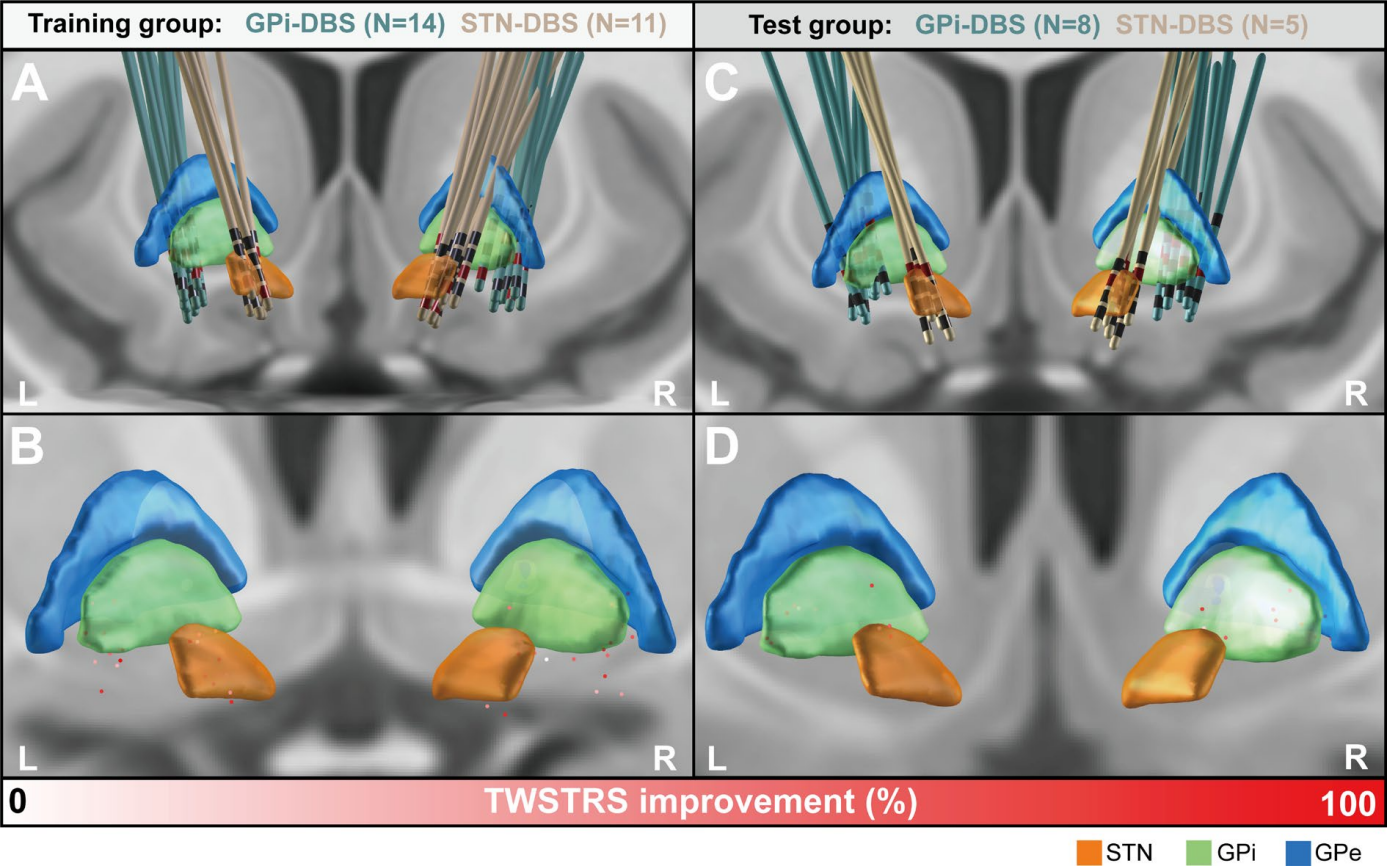
Reconstructions of DBS electrode implantation color‐coded by different targets. (A) The electrode position of GPi‐DBS and STN‐DBS in the training group, (B) the point cloud weighted by the improvement rate of TWSTRS score in the training group, (C) the electrode position of GPi‐DBS and STN‐DBS in the test group and (D) the point cloud weighted by the improvement rate of TWSTRS score in the test group.

The result of the test group is similar to that of the training group. Detailed information on patient characteristics at the group level and individual level can be found in Tables 1 and 2, respectively. The electrode positions of DBS lead and the point clouds weighted by the percentage improvement of TWSWRS score are shown in Figure 1A,B for the training group and Figure 1C,D for the test group, respectively. The VTA models intersecting with the STN and GPi, and GPe showed no significant association with clinical improvement (Figure S2).

**TABLE 1:** Clinical and demographical features of CD patients with GPi‐ or STN‐DBS at group level.

Training group	Total (*n* = 25)		GPi‐DBS (*n* = 14)		STN‐DBS (*n* = 11)		*p*
**Preoperative assessment**							
Sex (male:female)	12:13		3:11		9:2		**0.0027** ^**a**^
Age (year)	46.00 ± 12.08		47.50 ± 11.42		44.09 ± 13.16		0.4952^b^
TWSTRS total	47.09 ± 11.62		45.79 ± 13.20		48.75 ± 9.604		0.5381^b^
TWSTRS severity	21.92 ± 4.329		20.57 ± 3.817		23.64 ± 4.501		0.0783^b^
TWSTRS disability	17.28 ± 5.948		17.50 ± 6.607		17.00 ± 5.292		0.8398^b^
TWSTRS pain	8.290 ± 5.712		7.714 ± 6.005		9.023 ± 5.510		0.5806^b^
MoCA	26.80 ± 1.500		26.64 ± 1.277		27.00 ± 1.789		0.4956^c^
HAMA	8.480 ± 3.630		8.571 ± 3.204		8.364 ± 4.273		0.8905^b^
HRSD	11.60 ± 5.000		12.07 ± 5.298		11.00 ± 4.775		0.6055^b^
**Postoperative assessment**		***p*′**		***p*′**		***p*′**	
Follow‐up (month)	29.88 ± 16.52		37.29 ± 15.02		20.45 ± 13.68		**0.0057** ^c^
No. of electrodes	50		28		22		—
Pulse width (μs)	66.40 ± 8.751		67.50 ± 10.05		65.00 ± 6.726		0.5522^c^
Frequency (Hz)	136.9 ± 8.915		138.4 ± 10.98		135.0 ± 4.880		0.4433^c^
Voltage (V)	2.505 ± 0.5092		2.811 ± 0.3713		2.116 ± 0.3803		**< 0.0001** ^c^
TWSTRS total	18.44 ± 15.19		18.07 ± 15.42		18.91 ± 15.61		0.8945^b^
Improvement rate	0.6297 ± 0.2821	**< 0.0001** ^e^	0.6234 ± 0.3106	**< 0.0001** ^d^	0.6377 ± 0.2558	**< 0.0001** ^d^	0.9028^b^
TWSTRS severity	9.280 ± 7.289		8.857 ± 7.242		9.818 ± 7.666		0.7511^b^
Improvement rate	0.5874 ± 0.3002	**< 0.0001** ^d^	0.5816 ± 0.3285	**< 0.0001** ^d^	0.5947 ± 0.2756	**< 0.0001** ^d^	0.9166^b^
TWSTRS disability	8.200 ± 7.071		8.500 ± 6.813		7.818 ± 7.705		0.8166^b^
Improvement rate	0.5645 ± 0.3503	**< 0.0001** ^d^	0.5478 ± 0.3650	**0.0002** ^d^	0.5858 ± 0.3469	**0.004** ^d^	0.7938^b^
TWSTRS pain	0.7600 ± 2.368		0.7857 ± 2.424		0.7273 ± 2.412		0.8549^c^
Improvement rate	0.9241 ± 0.2450	**< 0.0001** ^e^	0.9358 ± 0.2013	**0.0020** ^e^	0.9091 ± 0.3015	**0.0078** ^e^	> 0.9999^c^
MoCA	27.12 ± 1.666		26.71 ± 1.437		27.64 ± 1.859		0.1747^b^
Improvement rate	0.01285 ± 0.05048	0.2460^d^	0.003530 ± 0.05012	0.8724^e^	0.02471 ± 0.05074	0.1522^d^	0.2966^c^
HAMA	6.320 ± 4.337		6.500 ± 4.848		6.091 ± 3.807		0.8206^b^
Improvement rate	0.2945 ± 0.2995	**0.0001** ^d^	0.2918 ± 0.3694	**0.0266** ^d^	0.2979 ± 0.1944	**< 0.0001** ^d^	0.9603^b^
HRSD	8.200 ± 5.831		9.286 ± 7.065		6.818 ± 3.601		0.4246^c^
Improvement rate	0.3083 ± 0.3589	**0.0018** ^**e**^	0.2363 ± 0.4524	0.1075^**e**^	0.4001 ± 0.1627	**< 0.0001** ^**d**^	0.9475^c^

Test group	Total (*n* = 13)		GPi‐DBS (*n* = 8)		STN‐DBS (*n* = 5)		*p*
**Preoperative assessment**							
Sex (male:female)	8:5		5:3		2:3		0.4285^a^
Age (year)	51.15 ± 12.03		50.38 ± 10.86		52.40 ± 14.99		0.7819^b^
TWSTRS total	34.85 ± 10.78		36.50 ± 9.024		32.20 ± 13.85		0.5080^b^
TWSTRS severity	19.69 ± 6.421		19.88 ± 6.833		19.40 ± 6.465		0.9033^b^
TWSTRS disability	12.62 ± 6.923		14.00 ± 6.719		10.40 ± 7.403		0.3847^b^
TWSTRS pain	2.538 ± 2.602		2.625 ± 2.925		2.400 ± 2.302		0.8870^b^
MoCA	26.85 ± 2.478		27.88 ± 0.9910		25.20 ± 3.347		0.06523^b^
HAMA	11.15 ± 7.175		14.00 ± 5.806		6.600 ± 7.301		0.0671^b^
HRSD	10.46 ± 5.254		12.13 ± 3.758		7.800 ± 6.611		0.1565^b^
**Postoperative assessment**		***p*′**		***p*′**		***p*′**	
Follow‐up (month)	24.00 ± 18.85		24.00 ± 19.51		14.40 ± 5.367		0.5377^c^
No. of electrodes	26		16		10		—
Pulse width (μs)	65.00 ± 8.602		66.88 ± 10.14		62.00 ± 4.216		0.2421^c^
Frequency (Hz)	137.3 ± 8.029		136.9 ± 9.979		138.0 ± 3.496		0.1549^c^
Voltage (V)	2.510 ± 0.6317		2.953 ± 0.2148		1.800 ± 0.3496		**< 0.0001** ^**c**^
TWSTRS total	12.92 ± 7.994		14.00 ± 8.106		11.20 ± 8.408		0.5621^b^
Improvement rate	0.6262 ± 0.2380	**< 0.0001** ^d^	0.6012 ± 0.2379	**0.0006** ^d^	0.6662 ± 0.2601	**0.0231** ^d^	0.6526^b^
TWSTRS severity	8.231 ± 4.986		9.000 ± 5.099		7.000 ± 5.099		0.5057^b^
Improvement rate	0.5533 ± 0.2716	**0.0001** ^d^	0.4962 ± 0.2735	**0.0082** ^d^	0.6446 ± 0.2710	**0.0120** ^d^	0.3600^b^
TWSTRS disability	3.846 ± 3.184		4.000 ± 3.546		3.600 ± 2.881		0.8365^b^
Improvement rate	0.6710 ± 0.2873	**0.0007** ^d^	0.6887 ± 0.2949	**0.0058** ^d^	0.6425 ± 0.3061	0.0925^d^	0.7913^b^
TWSTRS pain	0.8462 ± 1.214		1.000 ± 1.414		0.6000 ± 0.8944		0.8549^c^
Improvement rate	0.5621 ± 1.086	0.0547^e^	0.3696 ± 1.376	0.2500^e^	0.8700 ± 0.1857	0.2500^e^	0.6301^c^
MoCA	27.54 ± 1.898		28.38 ± 0.7440		26.20 ± 2.490		**0.0376** ^**b**^
Improvement rate	−0.02905 ± 0.05171	0.1250^e^	−0.01902 ± 0.04306	0.4063^e^	−0.04510 ± 0.06523	0.4063^d^	0.4351^c^
HAMA	8.000 ± 5.385		10.13 ± 3.682		4.600 ± 6.309		0.0688^b^
Improvement rate	0.3417 ± 0.3382	**0.0057** ^d^	0.2374 ± 0.2017	**0.0253** ^d^	0.5088 ± 0.4640	**0.1292** ^d^	0.1682^b^
HRSD	7.231 ± 4.622		9.750 ± 3.059		3.200 ± 3.834		**0.0057** ^b^
Improvement rate	0.3558 ± 0.3317	**0.0023** ^d^	0.2012 ± 0.1214	**0.0053** ^d^	0.6031 ± 0.4243	0.0766^d^	**0.0257** ^b^

*Note:* A *p* value < 0.05/3 ≈0.017 (Bonferroni correction) indicates statistical significance for the subscores of TWSTRS due to multiple comparisons. Data were presented in the form of mean ± SD and bold indicates statistical significance.

Abbreviations: CD, cervical dystonia; DBS, deep brain stimulation; GPi, the globus pallidus internus; HAMA, Hamilton Anxiety Scale; HRSD, Hamilton Rating Scale for Depression; MoCA, Montreal Cognitive Assessment; STN, the subthalamic nucleus; TWSTRS, Toronto Western Spasmodic Torticollis Rating Scale.

^a^ Fisher exact test.

^b^ Unpaired *t* test.

^c^ Mann–Whitney *U* test.

^d^ Paired *t* test.

^e^ Wilcoxon test.

**TABLE 2:** Clinical and demographic features of CD patients with GPi‐ or STN‐DBS at individual level.

ID	Target	Sex	Age at surgery (years)	Disease duration (years)	Type of CD	Follow‐up (months)	Preop TWSTRS total	Postop TWSTRS total	Programming, contact/pulse width (μs)/frequency (Hz)/voltage (V)	Preop oral medication	Postop oral medication	Previous BTX injections
Training group: Cohort 1 (*n* = 25, Beijing Tiantan Hospital)												
#01	GPi	F	53	1.25	LR + LC + RC	63	40	1	C+1‐ 90 140 2.95 C+5‐ 70 175 3.35	Baclofen, haloperidol, eperisone	None	Secondary non‐response
#02	GPi	F	63	6	LR + LC + RC	60	43.5	31	C+2‐ 80 140 2.7 C+6‐ 70 140 2.6	Baclofen, tiapride	None	None
#03	GPi	F	34	7	LC	60	56	1	C+2‐ 60 130 3.5 C+6‐ 60 130 3.5	Benzhexol, baclofen, clonazepam	None	Secondary non‐response
#04	GPi	F	48	1	RC	45	67.25	23	C+1‐ 90 160 2.6 C+5‐ 80 160 2.8	Benzhexol, baclofen, clonazepam	None	Secondary non‐response
#05	GPi	F	53	2	LR + LC + RC	24	59	55	C+2‐ 60 140 3.4 C+5‐ 60 140 3.6	Baclofen, clonazepam	Baclofen	Primary non‐response
#06	GPi	M	57	10	LR	24	46.75	18	C+3‐ 60 130 2.5 C+7‐ 60 130 2.7	Clonazepam	None	None
#07	GPi	F	31	10	RC	41	30	1	C+1‐ 60 135 2.8 C+5‐ 60 135 3.0	Baclofen, benzhexol	None	Secondary non‐response
#08	GPi	F	43	10	LR + LC + RC	44	54	19	C+2‐ 70 140 2.9 C+6‐ 70 140 2.8	None	None	Secondary non‐response
#09	GPi	F	39	5	AC	32	61.25	14	C+2‐ 80 130 2.7 C+6‐ 80 130 2.8	Baclofen, clonazepam	None	None
#10	GPi	F	66	1	LR	29	43.25	14	C+3‐ 70 140 2.6 C+7‐ 60 140 2.5	None	None	Secondary non‐response
#11	GPi	M	56	2.5	RC	28	44	28	C+1‐ 60 130 2.8 C+5‐ 60 130 2.4	None	None	Secondary non‐response
#12	GPi	F	50	2	LR	27	37	33	C+1‐ 60 130 2.5 C+5‐ 60 130 2.6	Benzhexol, baclofen, clonazepam	Clonazepam	None
#13	GPi	F	30	2	LR + LC + RC	18	17	1	C+2‐ 80 145 2.9 C+6‐ 60 145 2.0	Benzhexol, baclofen, clonazepam	None	Primary non‐response
#14	GPi	M	42	1.5	LR	27	42	14	C+1‐ 60 130 2.5 C+5‐ 60 130 2.7	None	None	Primary non‐response
#15	STN	M	34	10	LR + LC + RC	40	33	1	C+1‐ 60 140 2.0 8 + 5‐ 60 140 1.8	Benzhexol, baclofen, clonazepam	None	Secondary non‐response
#16	STN	M	43	2	LR	47	48	15	2 + 4‐ 80 140 2.5 6 + 8‐ 80 140 2.5	Clonazepam	Clonazepam	Secondary non‐response
#17	STN	M	31	15	LR	12	53.25	31	C+1‐ 60 130 2.0 C+5‐ 60 130 2.1	Baclofen, clonazepam	None	None
#18	STN	M	50	15	LR + RC	12	67	13	C+2‐ 60 130 1.8 C+6‐ 60 130 1.85	None	None	Secondary non‐response
#19	STN	M	28	2.5	LR + LC + RC	12	45	8	C+1‐ 70 140 2.9 C+5‐ 70 140 3.0	None	None	Secondary non‐response
#20	STN	M	24	4	LR	12	39	3	C+4‐ 60 130 2.0 C+8‐ 60 130 2.2	Benzhexol, baclofen	None	Secondary non‐response
#21	STN	F	54	1.9	LR + AC	12	51	18	C+2‐ 60 130 1.8 C+7‐ 60 130 1.5	Benzhexol, baclofen	None	None
#22	STN	M	49	1.3	LR	34	46	20	C+2‐ 60 130 2.1 C+6‐ 60 130 1.6	Clonazepam	None	None
#23	STN	M	57	23	LR	15	44	18	C+4‐ 70 140 2.6 C+8‐ 60 140 2.0	None	None	Secondary non‐response
#24	STN	M	65	6	LC	23	62	58	C+2‐ 70 135 2.2 C+6‐ 70 135 2.0	Benzhexol, baclofen, clonazepam	Benzhexol, baclofen, clonazepam	Secondary non‐response
#25	STN	F	50	5	LR	6	48	23	C+1‐ 70 140 2.1 C+5‐ 70 140 2.0	Benzhexol, baclofen, clonazepam	None	Secondary non‐response
Test group: Cohort 2 (*n* = 6, Beijing Fengtai Hospital)												
#26	GPi	F	64	2.5	LR	48	36	4	C+3‐ 60 140 2.8 C+7‐ 60 140 2.7	Baclofen, clonazepam	None	Secondary non‐response
#27	GPi	M	38	3	RC	24	51	19	C+2‐ 60 130 2.8 C+6‐ 60 130 3.0	Clonazepam	None	Primary non‐response
#28	STN	F	28	5	AC	24	40	17	C+3‐ 60 130 3.0 C+7‐ 60 130 1.5	Benzhexol, baclofen	None	None
#29	STN	M	53	8	LR + LC + RC	12	49	6	C+3‐ 90 135 2.0 C+7‐ 90 135 1.5	Clonazepam	None	Secondary non‐response
#30	STN	F	69	10	LR	12	23	12	C+4‐ 60 140 1.5 C+8‐ 60 140 1.5	None	None	Secondary non‐response
#31	STN	M	55	2	LR + LC + RC	12	14	3	C+3‐ 90 130 2.1 C+8‐ 90 130 1.8	None	None	Secondary non‐response
Test group: Cohort 3 (*n* = 5, The First Affiliated Hospital of Soochow University)												
#32	GPi	M	44	2.25	RC	60	23	9	C+3‐ 80 160 3.0 C+7‐ 80 160 3.0	None	None	Secondary non‐response
#33	GPi	M	51	3	LR + LC + RC	12	42	27	C+2‐ 60 130 3.4 C+6‐ 60 130 3.4	Benzhexol, baclofen, clonazepam	Clonazepam	Unknown
#34	GPi	M	48	3	LR	12	38	16	C+2‐ 60 130 2.75 C+6‐ 60 130 2.8	None	None	Secondary non‐response
#35	GPi	F	51	10	RC	6	25	20	C+2‐ 60 130 2.8 C+6‐ 60 130 2.8	Benzhexol, baclofen, clonazepam	Clonazepam	Secondary non‐response
#36	GPi	F	39	12	RC	12	37	13	C+4‐ 90 130 2.7 C+8‐ 90 130 2.4	Clonazepam	None	Secondary non‐response
Test group: Cohort 4 (*n* = 2, Weihai Central Hospital)												
#37	GPi	M	68	7	LR + LC + RC	18	40	4	C+2‐ 60 130 2.7 C+6‐ 60 130 2.75	Clonazepam	None	Secondary non‐response
#38	STN	F	57	4	LR + LC + RC	12	35	21	C+4‐ 70 140 2.2 C+8‐ 70 140 2.4	Benzhexol, baclofen	None	None

Abbreviations: AC, anterocollis; BTX, botulinum toxin; CD, cervical dystonia; DBS, deep brain stimulation; GPi, the globus pallidus internus; LC, laterocollis; LR, latero‐rotatory; RC, retrocollis; STN, the subthalamic nucleus; TWSTRS, the Toronto Western Spasmodic Torticollis Rating Scale.

In addition, no patient suffered from surgery‐ or device‐related adverse events (AEs) during the follow‐up period. Regarding stimulation‐related AEs, three patients in the GPi‐DBS group experienced dysarthria, and two patients in the STN‐DBS group had dyskinesia. However, these stimulation‐related AEs were transient, and symptoms disappeared after programming.

### 3.2 PSMs

**FIGURE 2:**
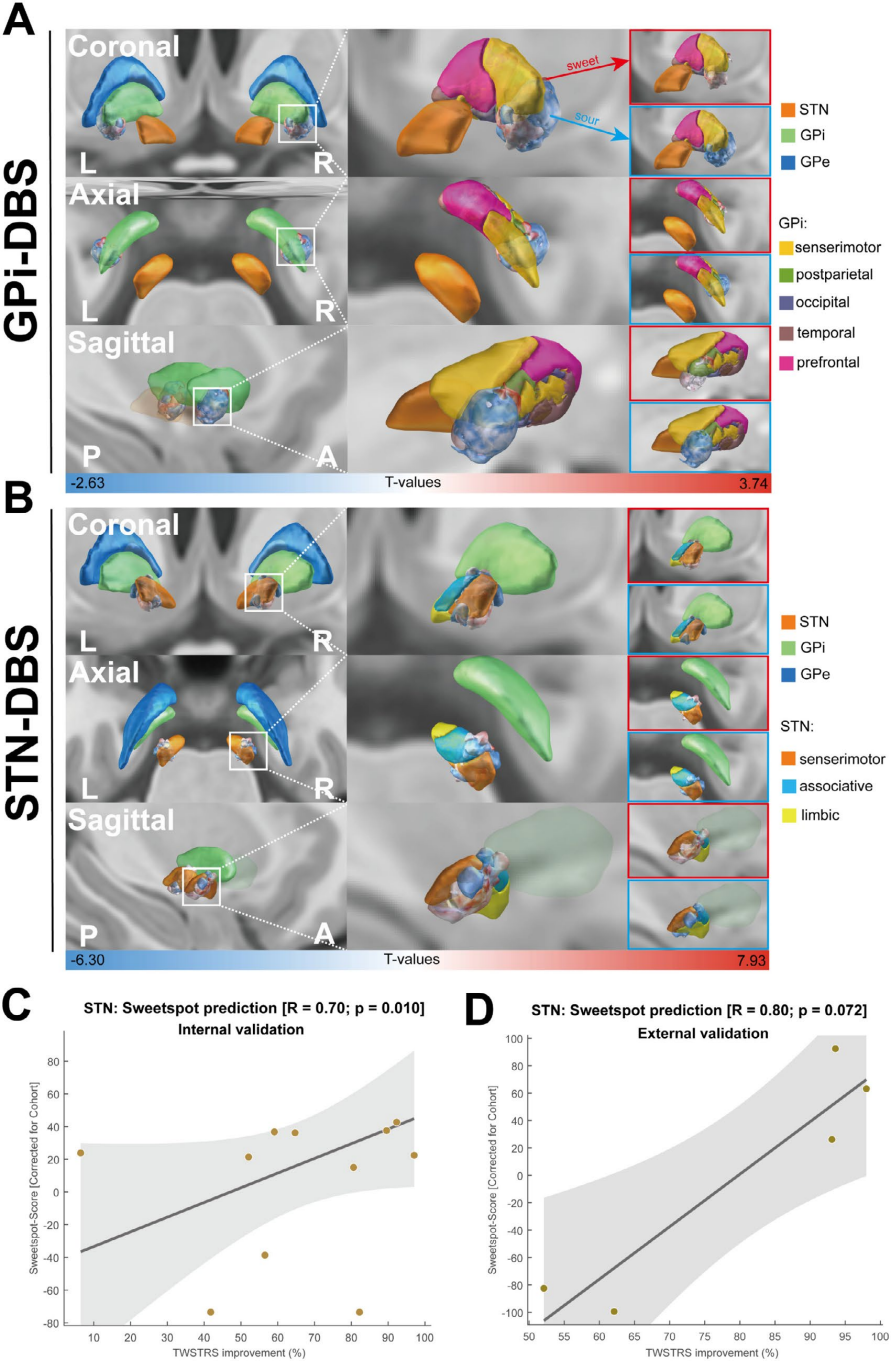
Sweet spot (red) and sour spot (blue) maps of changes in TWSTRS score in (A) GPi‐DBS, (B) STN‐DBS. Voxels are color‐coded based on degree of correlation between improvement (hot colors) or deterioration (cool colors) of the TWSTRS score and are shown as coronal (upper), axial (middle), and sagittal (lower) views. The degree of how fittingly the identified probabilistic stimulation map for the STN‐DBS cohort in (C) internal validation and (D) external validation.

The results of PSMs are shown in Figure 2. For GPi‐DBS, the posterior ventral medial GPi, together with a small part of the posterior lateral GPi, was identified as optimal or “sweet spots” for improvement of TWSTRS scores, which were mainly distributed in the sensorimotor part of the GPi; while the “sour spot” associated with suboptimal benefit was distributed in the GPe and lateral side of the GPi, and was located in the lateral side of the sweet spot (Figure 2A). Regarding STN‐DBS, the sweet spot for the ΔTWSTRS score was located in the dorsolateral part of the STN, which mainly covered the sensorimotor system, whereas the sour spot for ΔTWSTRS was situated on the lateral side and shifted to the ventral medial side of the STN, occupying the sensorimotor and associative STN (Figure 2B). The sweet spot model of GPi‐DBS failed to predict clinical improvements (Figure S3A), whereas the model of STN‐DBS significantly predicted improvements in TWSTRS scores in internal validation (*R*_*i*_ = 0.70, *p*_*i*_ = 0.010; Figure 2C) but failed in external validation (*R*_e_ = 0.80, *p*_e_ = 0.072; Figure 2D). The result of the models did not change significantly after adjusting covariates (Figure S4), and sensitivity analysis based on ΔTWSTRS_severity_ produced similar results to the main analysis (Figure S5).

### 3.3 Structural Connectivity Analysis

**FIGURE 3:**
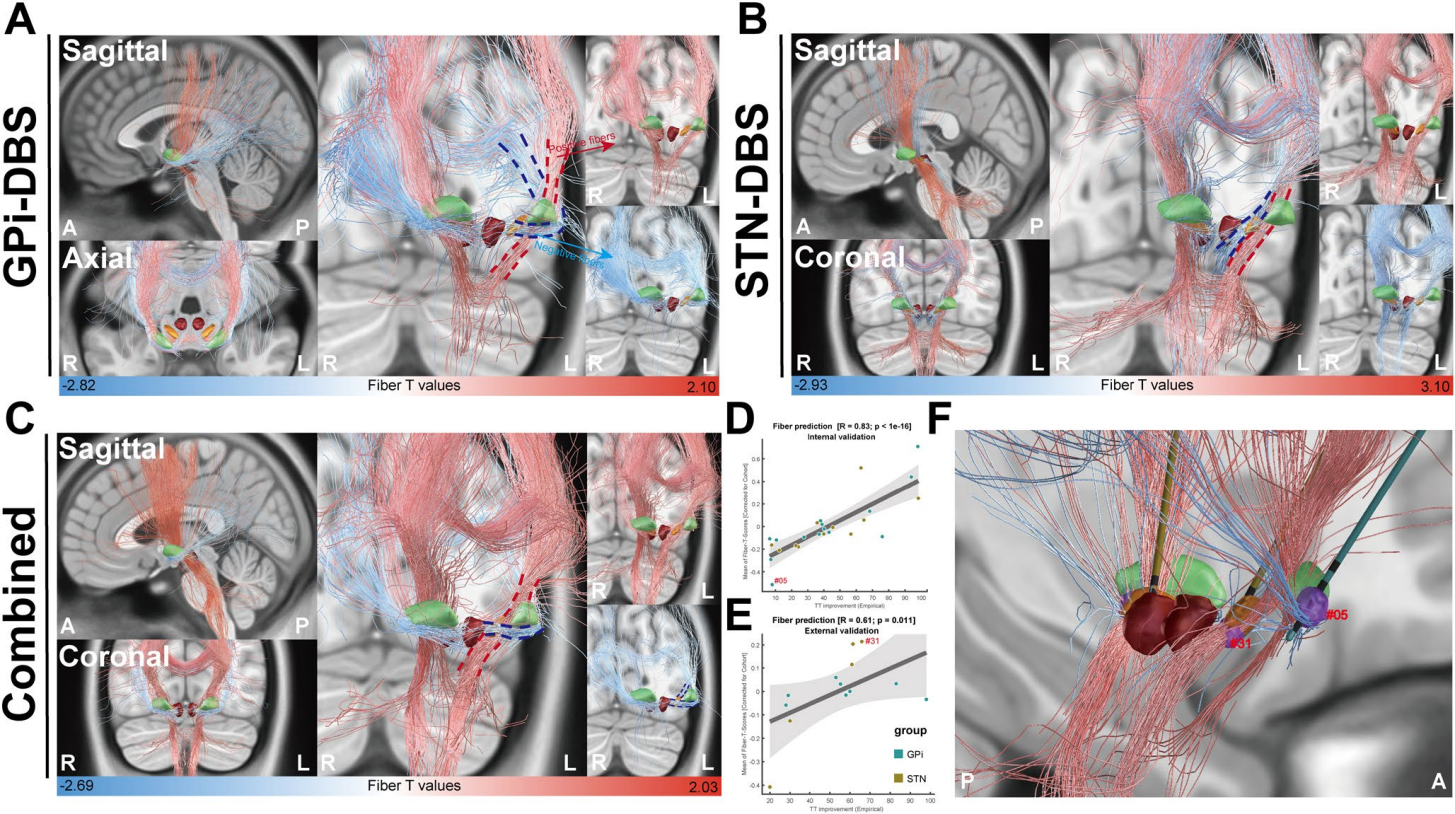
Reconstruction of fiber tracts associated with optimal improvement of TWSTRS score in (A) GPi‐DBS, (B) STN‐DBS, and (C) the combined dataset. Red fiber tracts are positively linked to improvements in TWSTRS score with positive fiber *T*‐scores, whereas blue fiber tracts are negatively linked to improvements in TWSTRS score with negative fiber *T*‐scores. The degree of how fittingly the identified structural connectivity model for the combined dataset in (D) internal validation and (E) external validation. (F) The representative picture of VTAs for two patients with different clinical improvement (#05: GPi‐DBS patient with poor improvement in the training group; #31: STN‐DBS patient with good improvement in the test group) and ideal structural connectivity model.

Figure 3 shows the structural connectivity analyses of the two targets that were used to determine the number of fiber tracts associated with ΔTWSTRS scores. In the GPi‐DBS cohort, positive fibers associated with optimal clinical outcomes (red fibers) projected mainly to the primary motor cortex (M1), whereas a few fibers projected to somatosensory cortices (including the somatosensory and somatosensory association cortices) and cerebellum. In contrast, negative fibers associated with poor clinical outcomes (blue fibers) projected mainly to occipital cortices (including primary and association visual cortices). In addition, positively associated fibers of GPi‐DBS projected to the cortex by traversing the posterior comb system, whereas negatively associated fibers passed through the base of the GPi and were projected to the cortex through the dorsal lateral side of positively associated fibers (Figure 3A). In the STN‐DBS cohort, positively associated fibers were projected mainly to the M1 region, premotor cortices (including the premotor cortex [PMC] and supplementary motor area [SMA]), and cerebellum. Regarding negatively associated fibers in STN‐DBS, they also projected to the M1 region and premotor cortices (Figure 3B). The positively (lateral side) and negatively (medial side) associated fiber tracts in STN‐DBS traversed through different pathways at the basal ganglia level and intersected at the cortical level (Figure 3B). For the combined dataset, the fibers projecting to the premotor cortices, M1, dorsolateral prefrontal cortex (dlPFC), and cerebellum seemed beneficial to ΔTWSTRS score. In contrast, it was negatively associated with tracts to the somatosensory and visual cortices (Figure 3C). According to leave‐one‐out internal validation, there was no statistical significance in the structural connectivity model of GPi/STN‐DBS (Figure S3B,C). However, a significant prediction was observed in the structural connectivity model of the combined dataset (*R*_*i*_ = 0.83, *p*_*i*_ < 1 × 10^−16^; *R*_e_ = 0.61, *p*_e_ = 0.011; Figure 3D,E), and the relationship of VTAs and ideal structural connectivity model for two typical patients with different clinical improvement is shown in Figure 3F. In addition, intersection analysis was conducted to detect associations with VTAs and different brain regions. The results are similar to those of structural connectivity analysis (Figure S6). Moreover, no significant difference was observed after adjusting covariates in the connectivity analysis (Figure S7), and sensitivity analysis using ΔTWSTRS_severity_ showed similar results to the main analysis (Figure S8).

### 3.4 Functional Connectivity Analysis

**FIGURE 4:**
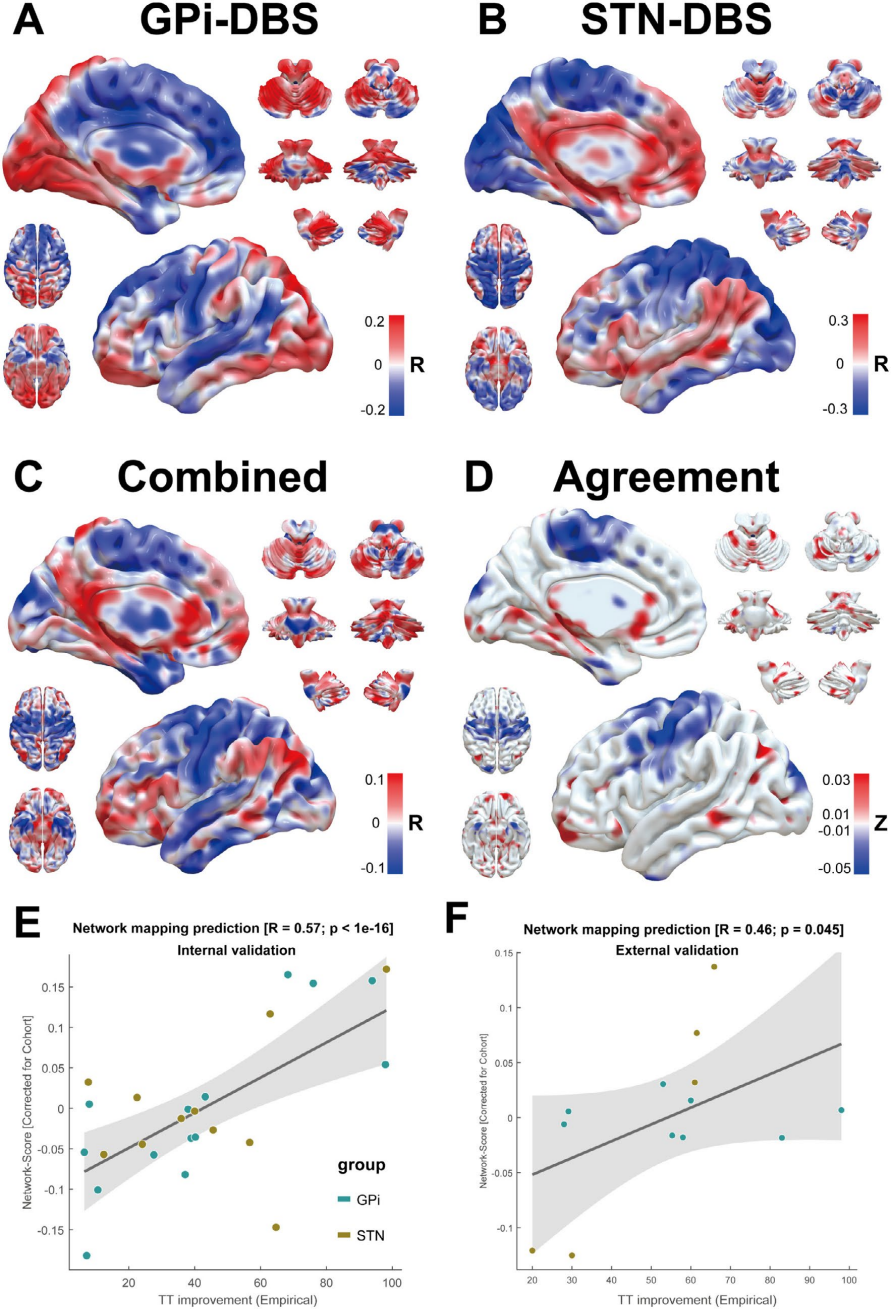
Functional connectivity profile associated with optimal improvement of TWSTRS score. R‐map from (A) GPi‐DBS and (B) STN‐DBS, (C) the combined dataset, and (D) agreement map based on the GPi and STN‐DBS. Red regions showed connections positively correlated with clinical improvements, whereas blue regions showed connections negatively associated with clinical improvements. The degree of how fittingly the identified functional connectivity model for the combined dataset in (E) internal validation and (F) external validation.

As shown in Figure 4, improvements with GPi‐DBS were associated with positive connections (warm colors) to the orbitofrontal area, inferior prefrontal gyrus, supramarginal gyrus, visual cortices, somatosensory association cortex, and cerebellum (Figure 4A). In contrast, in STN‐DBS, the same was true for the dorsolateral PMC, frontopolar area, inferior prefrontal gyrus, angular gyrus, supramarginal gyrus, cingulate gyrus, low and middle temporal gyrus, and cerebellum (Figure 4B). The combined dataset favored positive connections to the PMC, frontopolar area, orbitofrontal area, angular gyrus, supramarginal gyrus, and cerebellum (Figure 4C). Finally, we constructed an agreement map to detect independent positively or negatively associated regions in GPi‐DBS and STN‐DBS (Figure 4D). The orbitofrontal area, superior temporal gyrus, angular gyrus, and cerebellum were positively connected to optimal clinical outcomes. In contrast, the SMC, temporopolar area, and visual association cortex were negatively associated with improvements in the TWSTRS scores. Although the functional model of GPi/STN‐DBS failed to predict clinical improvement using leave‐one‐out internal validation (Figure S3D,E), network mapping of the combined dataset also predicted significant clinical improvements of CD (*R*_*i*_ = 0.57, *P*_*i*_ < 1 × 10^−16^; *R*_e_ = 0.46, *P*_e_ = 0.045; Figure 4E,F). There was no significant difference after adjusting covariates or using ΔTWSTRS_severity_ as an indicator in the functional connectivity analysis (Figures S9 and S10).

## 4 Discussion

Our study identified the clinically derived stimulation regions, streamlines, and functional networks associated with clinical improvement in GPi/STN‐DBS for CD. It showed that the sweet spots of GPi and STN‐DBS were mainly located in the posterior ventral medial part of the GPi and dorsolateral part of the STN, respectively. Sweet‐spot mapping could significantly predict outcomes in the STN‐DBS cohort. In addition, the structural and functional connectivity analysis identified improvement/deterioration‐related fiber tracts and brain regions, which demonstrated similarities and differences between the two targets. Specifically, although the structural/functional connectivity maps generated based on the GPi and STN cohorts were not identical, we found that both targets engaged beneficial streamlines projecting to the M1 region and cerebellum, and the agreement map of functional connectivity exhibited several overlapping brain regions associated with optimal clinical outcomes. Finally, a model using optimal structural and functional connectivity successfully predicted the clinical improvement of CD patients with internal and external validation. This allowed us to predict the postoperative improvement of CD patients who underwent GPi/STN‐DBS surgeries based on a unified structural/functional connectivity model.

Although GPi‐DBS is a proven treatment for refractory CD, STN has emerged as another promising target, with the advantage of improved visualization during surgical planning and decreased power required for stimulation. However, there remains controversy about the optimal target for CD. Although patients may have varied responses to stimulation, they all experienced a degree of reduction in TWSTRS scores. Moreover, no statistical difference was found between the two targets in TWSTRS total score and subscores, which was consistent with a meta‐analysis conducted by Tsuboi et al. [10]. Using univariate regression analysis, we found that earlier age at surgery showed an association with improvements in TWSTRS scores, which was consistent with the conclusions from other studies [7, 10]. An explanation for the decreased efficacy of DBS with increased age is that plasticity may have been influenced by changes in synaptic functions associated over time [35].

Since CD is a movement disorder associated with dysfunction of motor or sensory cortico‐basal ganglia networks [36], restoring these networks is considered a goal when treating CD. The optimal target of GPi‐DBS associated with motor improvement has been studied in GPi‐DBS patients. Raghu et al. concluded that the posterior GPi limb of the cortico‐basal ganglia loop was associated with clinical improvement of CD [36]. In addition, Patriat et al. also confirmed that the motor region was generally distributed in the posterior part of the GPi and shifted to the posterolateral side, which was consistent with our stimulation contacts modulating symptoms [37]. Conversely, the sweet spot of STN‐DBS in CD has yet to be reported. The size of the STN is small, and the anatomical boundary is not well‐defined, indicating that functional areas may overlap in the STN [38]. Our findings suggested that the sweet spot for the ΔTWSTRS score was located in the dorsolateral STN, overlapping the sensorimotor part. This finding was consistent with the report by Yin et al., which suggested that the ideal implantation location was the central part of the dorsal STN [39]. Notably, the sweet spot for CD was similar to that for Parkinson's disease (PD), as reported by Dembek et al. [40]. A previous study observed that PD and dystonia demonstrated overlapping physiological characteristics, particularly regarding enhanced synchronization in motor cortical activity, with both conditions showing decreased cortical synchronization following STN‐DBS, which may explain the similar therapeutic response to basal ganglia stimulation [41]. However, similarities and minor differences in sweet spots for CD and PD should be further investigated.

In the structural connectivity of GPi‐DBS, most positively associated fibers were projected to the M1 region through the basal ganglia–thalamo–cortical network. In contrast, negatively related fibers were projected to the visual and somatosensory cortices. The role of M1 in the motor improvement of CD has been reported in studies showing that GPi‐DBS could regulate the excitability and plasticity of M1 [36]. In addition, we also found that these fibers projected to the cortex by traversing the posterior comb system, which was consistent with results reported by Horn et al. [16]. The posterior comb system based on the striatopallidofugal system plays an essential role in structural connectivity associated with clinical improvement [16]. For STN‐DBS, positive structural connectivities were projected to the M1, PMC, SMA, and cerebellum. Notably, STN‐DBS might better regulate cortical activities through cerebello–thalamo–cortical circuits, as more fibers traversed through the cerebellum in the STN group, which was also confirmed by functional connectivity [6, 41]. Recently, the SMA was found to be linked to postural control [43], and it was hypothesized that activation of the SMA may occur through the activation of fiber tracts in the hyperdirect pathway [44]. However, we could not characterize this pathway using our model; future studies targeting SMA may reveal the impact of structural connectivities on postural improvement in CD. Regarding the difference in structural connectivities between the two targets, Lai et al. reported that GPi and STN‐DBS may share a similar connection to the cortex because many fibers to the STN pass through the GPi [45]. However, whether this observation holds true in patients with CD remains to be determined. There were common efficacious streamlines (projections to M1 and cerebellum) in STN‐DBS and GPi‐DBS, which were more apparent in the combined cohort, and these results also predicted significant clinical improvements. Moreover, our findings also suggested STN‐DBS may have more influence on the PMC and SMA regions as it could affect a broader range of brain areas than GPi‐DBS [46].

Although studies on movement disorders have focused on the basal ganglia, the results of an increasing number of anatomical and pathophysiological studies have reported that the cerebellum plays an essential role in the clinical symptoms of CD [14]. In a health‐controlled study, CD patients showed increased functional connectivities between the motor cortex and cerebellum, which may have a compensatory effect on clinical symptoms [47]. At the whole‐brain level, Corp et al. reported that the optimal localization of GPi‐DBS was functionally connected to the cerebellum [6]. Our R‐map demonstrated similar results in both targets, confirming the mechanism of miscommunication between the basal ganglia and cerebellar loops during the progression of CD [6]. According to the R‐map, visual cortices were negatively connected to clinical improvements in STN‐DBS. The abnormalities of the visual cortex have been observed in neurophysiological studies and are thought to relate to a compensatory mechanism involving the CD head position [48]. The PMC, frontopolar area, inferior prefrontal gyrus, and cingulate gyrus were associated with improved clinical outcomes. These regions are responsive to STN‐DBS stimulation because of their direct connection to the STN [49], suggesting that STN‐DBS regulates these sites when modulating symptoms in CD. In addition, our study also showed that several frontal and temporal related regions played an essential role in DBS response. The metabolic changes of these regions in dystonia have also been observed [50, 51, 52]. For example, reorganizing the inferior frontal gyrus plays a role in compensatory mechanisms associated with the inhibition process in focal dystonia [53]. In the combined dataset, we found that the connectivities between stimulation sites and multiple cortical regions explained the clinical improvement because the R‐map in the combined dataset showed a significant predictive effect on the clinical outcome of CD. Our findings suggested that therapeutic brain stimulation could modulate brain networks rather than target individual brain areas [13, 38, 54, 55, 56]. In the network localization analysis conducted by Corp et al., the cerebellum and somatosensory cortices were positively and negatively connected, respectively, with optimal GPi‐DBS electrode locations [14]. Our study confirmed their findings and also detected the optimal functional connectivity of STN‐DBS. Both targets demonstrated overlapping efficacious functional connectivity in the cerebellum and somatosensory cortices, indicating that there may exist shared positive or negative influences on clinical improvement between the two targets. Overall, we suggest that these brain regions associated with clinical responses in DBS patients may also be potential therapeutic targets for non‐invasive stimulation. Koch et al. reported the efficacy of transcranial magnetic stimulation in the cerebellum in CD patients [57]. However, further studies on stimulation in brain regions with shared neuroanatomical networks are still needed.

This study had several limitations. First, the two targets follow‐up durations were statistically different, which may result in discrepant improvement in the TWSTRS scores. Second, although we minimized the inaccuracies induced by nonlinearly warping electrode sites into the template space, poor intrinsic precision still existed due to manual registration refinements [58]. Third, our study was conducted in cohorts with a relatively small sample size. The limited number of CD patients may cause biases and may hinder the translation of our findings to clinical practice despite the robustness tested by internal and external validation. Finally, our results were based on the normative map, including structural and functional connectivity, and acknowledged the patient‐specific differences in brain circuitry in CD patients.

## 5 Conclusion

In conclusion, we identified optimal stimulation sites and structural and functional connectivities associated with clinical improvements of GPi‐DBS and STN‐DBS in CD patients. We highlighted the differences and identified commonalities between the two targets. In addition, the combined dataset predicted clinical improvement of CD patients using structural and functional connectivity analyses, which indicated that a shared neuroanatomical network might exist between the two targets. Our findings have therapeutic implications for refining stimulation targets on specific brain regions.

## Author Contributions

Conceptualization: T.X., W.T., Y.B., and J.Z. Data curation: W.T., S.F., H.F., M.Y., and L.S. Formal analysis and validation: T.X., Y.Q., C.H., and T.N. Writing: T.X., Y.Q., and M.X. Critically revising the article: H.X., J.G., A.B., J.T., A.M.L., and J.Z. Methodology: Y.Q. and H.Z. Resource/technical/material: J.Z., A.Y., F.M., and Z.W. Funding acquisition: J.Z., Y.B., and H.X. Project administration and visualization: J.Z. F.G.M., Y.B., and J.Z. have directly accessed and verified the underlying data reported in the manuscript. All authors have full access to all the data in the study and accept responsibility to submit for publication.

## Ethics Statement

This retrospective study was approved by the Institutional Review Boards of Beijing Tiantan Hospital, registration number KY2022‐006‐02. This study took a retrospective approach, utilizing patient information from the medical record system. All participants provided written informed consent.

## Conflicts of Interest

The authors declare no conflicts of interest.

## Supporting information


**Data S1:** cns70561‐sup‐0001‐Supinfo.docx.

## Data Availability

The authors declare that all enrolled data can be found in the manuscript and Supporting Information.
