# Supplementary material for: Optimal Stimulation Sites and Connectomes for GPi and STN‐DBS in Cervical Dystonia
Source: CNS Neurosci Ther. 2025 Aug 17;31(8):e70561. doi: 10.1111/cns.70561 (PMC12358681; doi:10.1111/cns.70561)
Supplement: Supplementary file 1 — Data S1: cns70561‐sup‐0001‐Supinfo.docx. [file CNS-31-e70561-s001.docx]

**Optimal Stimulation Sites and Connectomes for GPi- and STN-DBS in Cervical Dystonia**

**Supplemental Data**

**Supplemental Figure 1:** Analysis workflow. Postoperative imaging was co-registered to preoperative MRI (**A**) and normalized to standard space following lead localization (**B**). VTAs were calculated (**C**) and assigned to the respective clinical outcome to conduct sweet/sour spot (**D**), structural connectivity (**E**) and functional connectivity(**F**) models based on training group. Internal validation **(G)** of three models were performed and only models with good predictive outcome were employed to perform external validation **(H)** by using test group data.


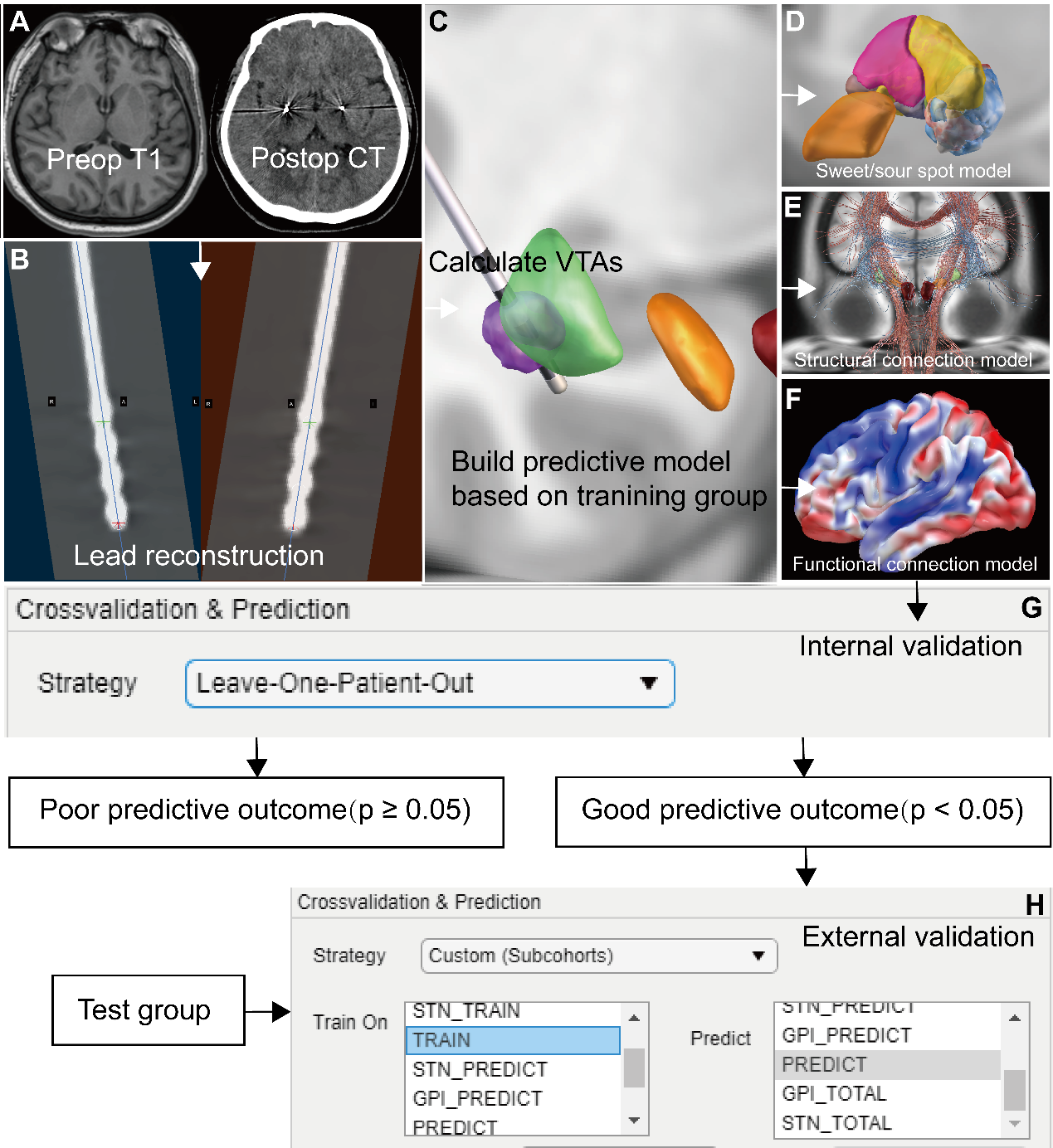


**Supplemental Figure 2:** The volume of tissue activated (VTA) of GPi-DBS intersections with the GPi (**A**), the sensorimotor part of GPi (**B**) and GPe (**C**); The volume of tissue activated (VTA) of STN-DBS intersections with the STN (**D**) and the motor part of STN (**E**).


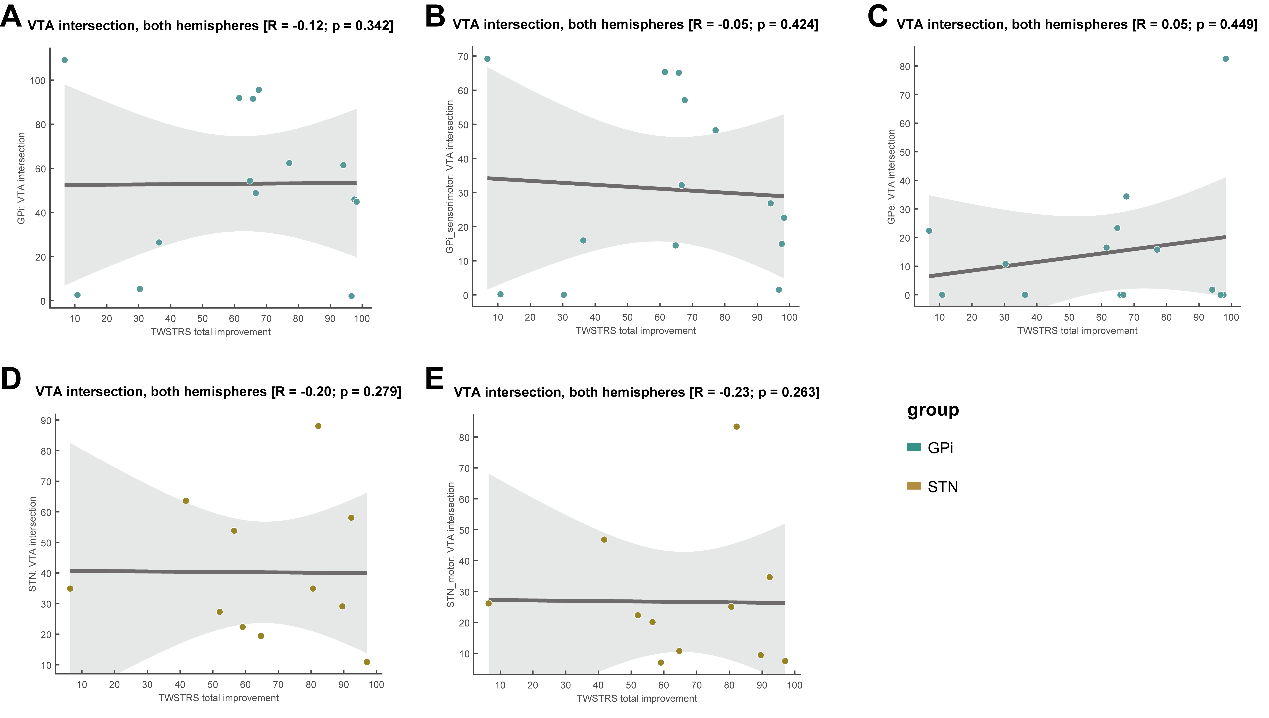


**Supplemental Figure 3:** The degree of how fittingly the identified probabilistic stimulation map (**A** for GPi-DBS ), structural connectivity (**B** for GPi-DBSm and **C** for STN-DBS) and functional connectivity models (**D** for GPi-DBS and **E** for STN-DBS) were modulated by each patient's volumes of tissue activated (VTAs) correlated with improvement rate of TWSTRS total scores though a leave-one-patient-out strategy (internal validation).


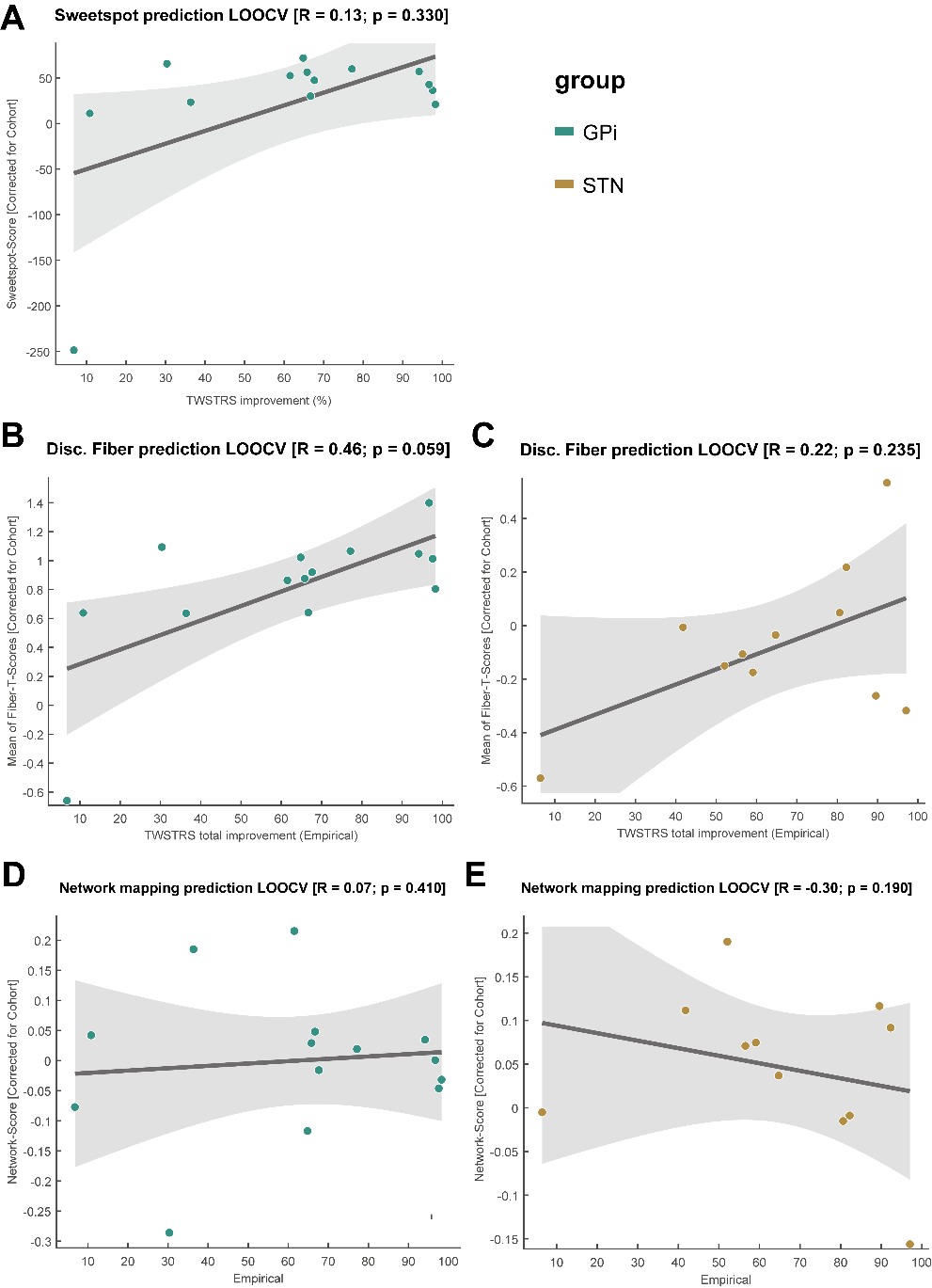


**Supplemental Figure 4:** Sweet spot (red) and sour spot (blue) maps of changes in TWSTRS total score in GPi and STN-DBS after adjusting covariates (such as sex, age at surgery, pre HAMA scores, pre HRSD scores, and pre MoCA scores).


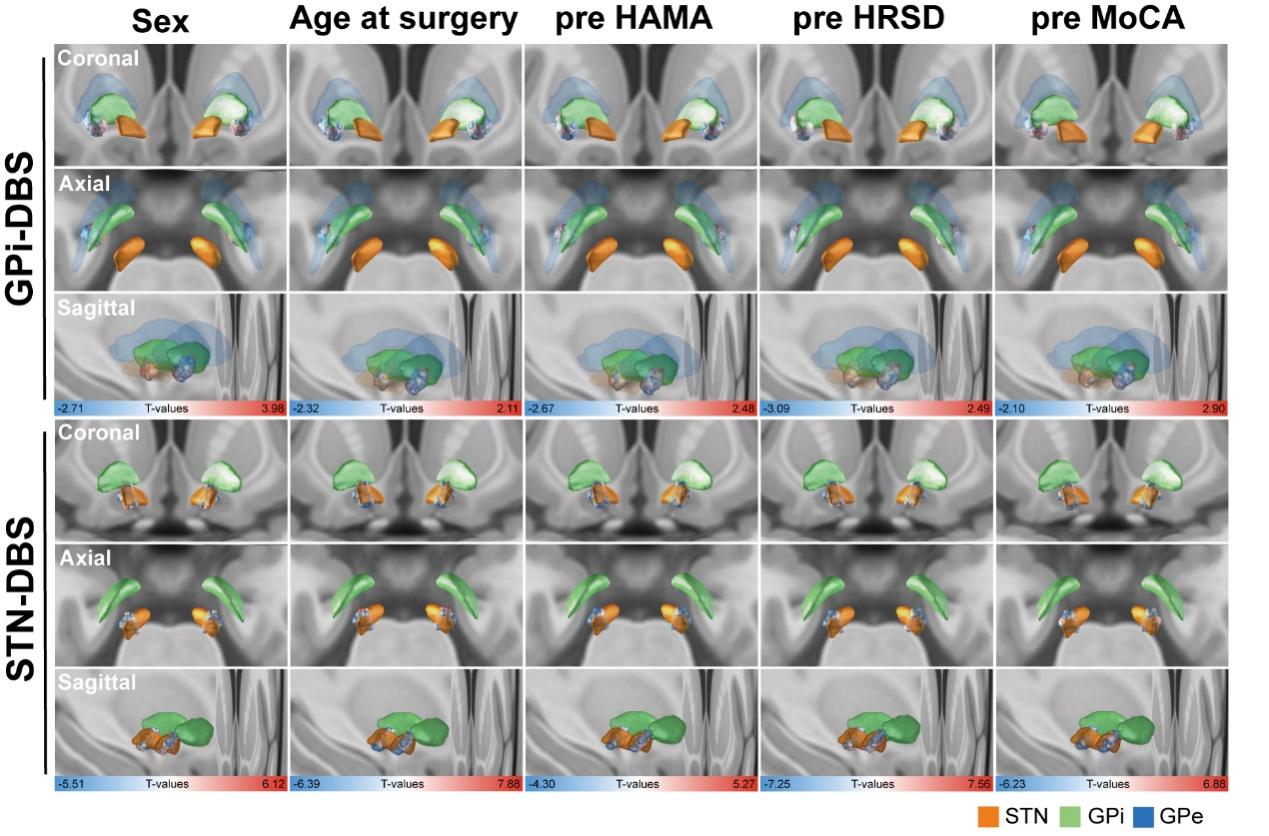


**Supplemental Figure 5:** Sweet spot (red) and sour spot (blue) maps of changes in TWSTRS severity score in GPi and STN-DBS.


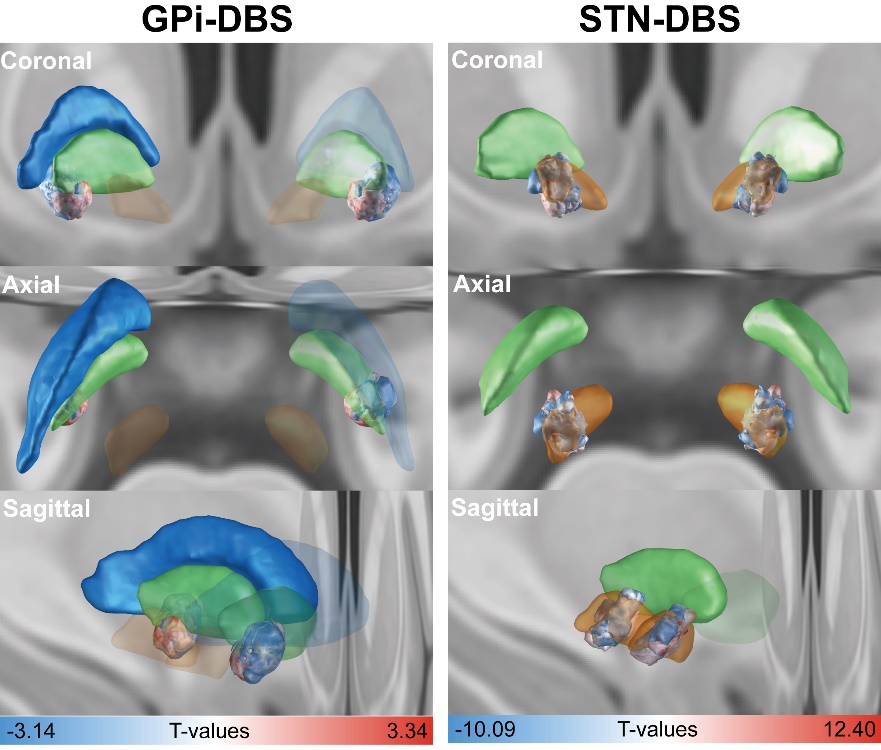


**Supplemental Figure 6:** The fiber connections from the VTAs of GPi-DBS to the TWSTRS total improvement–related regions: middle frontal gyrus (**A**), cerebellum (**B** and **C**) and TWSTRS total deterioration–related regions: superior occipital gyrus (**D**), superior temporal gyrus (**E**) and temporal pole (**F**). The fiber connections from the VTAs of STN-DBS and combined data to the TWSTRS total improvement–related regions: cerebellum (**G**, **H** for STN-DBS; **I**, **J** for combined data) The gray areas represent the 95% confidence bands of the best fit line.


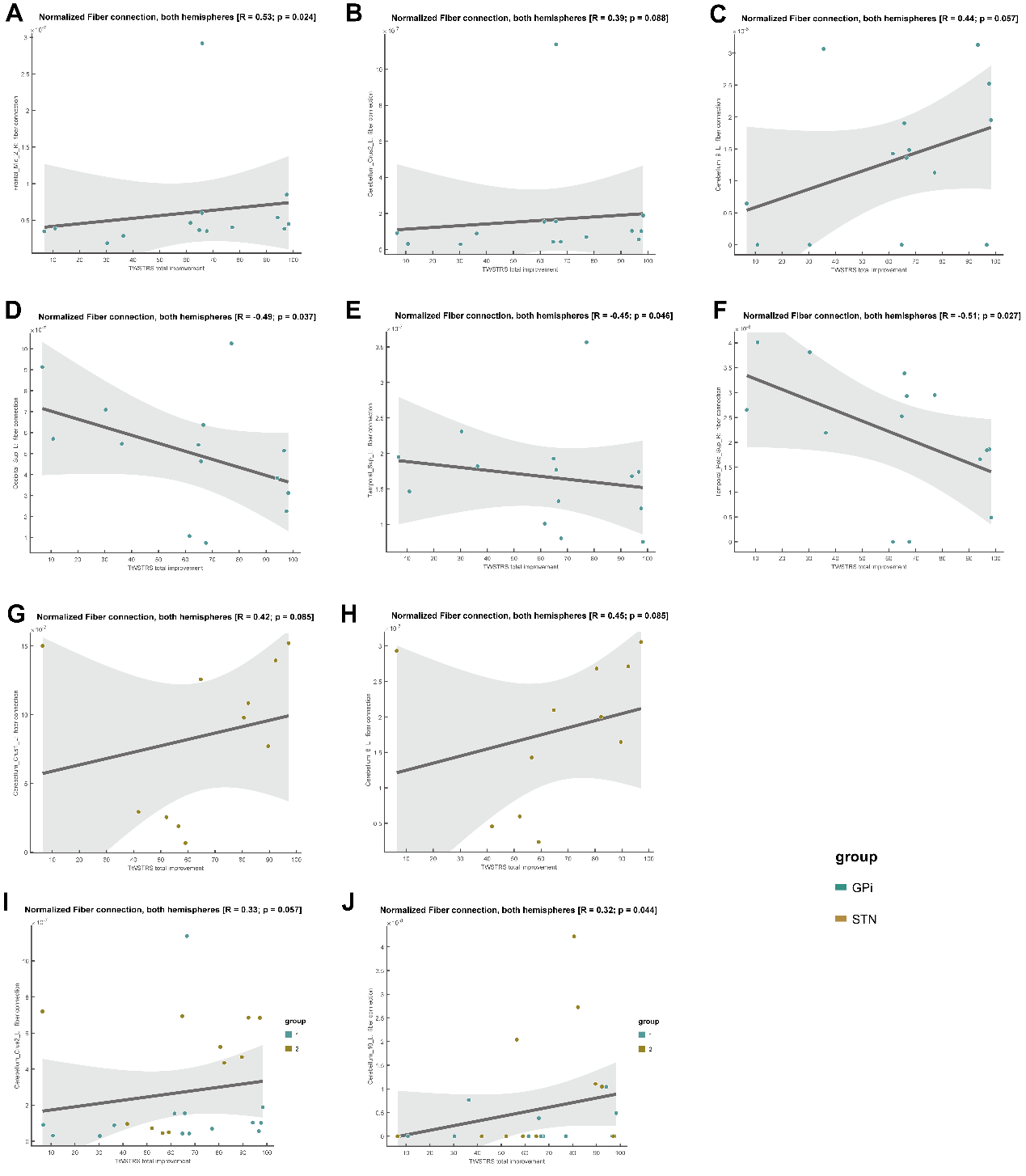


**Supplemental Figure 7:** Reconstruction of fiber tracts associated with optimal improvement of TWSTRS total score in GPi and STN-DBS after adjusting covariates (such as sex, age at surgery, pre HAMA scores, pre HRSD scores, and pre MoCA scores).


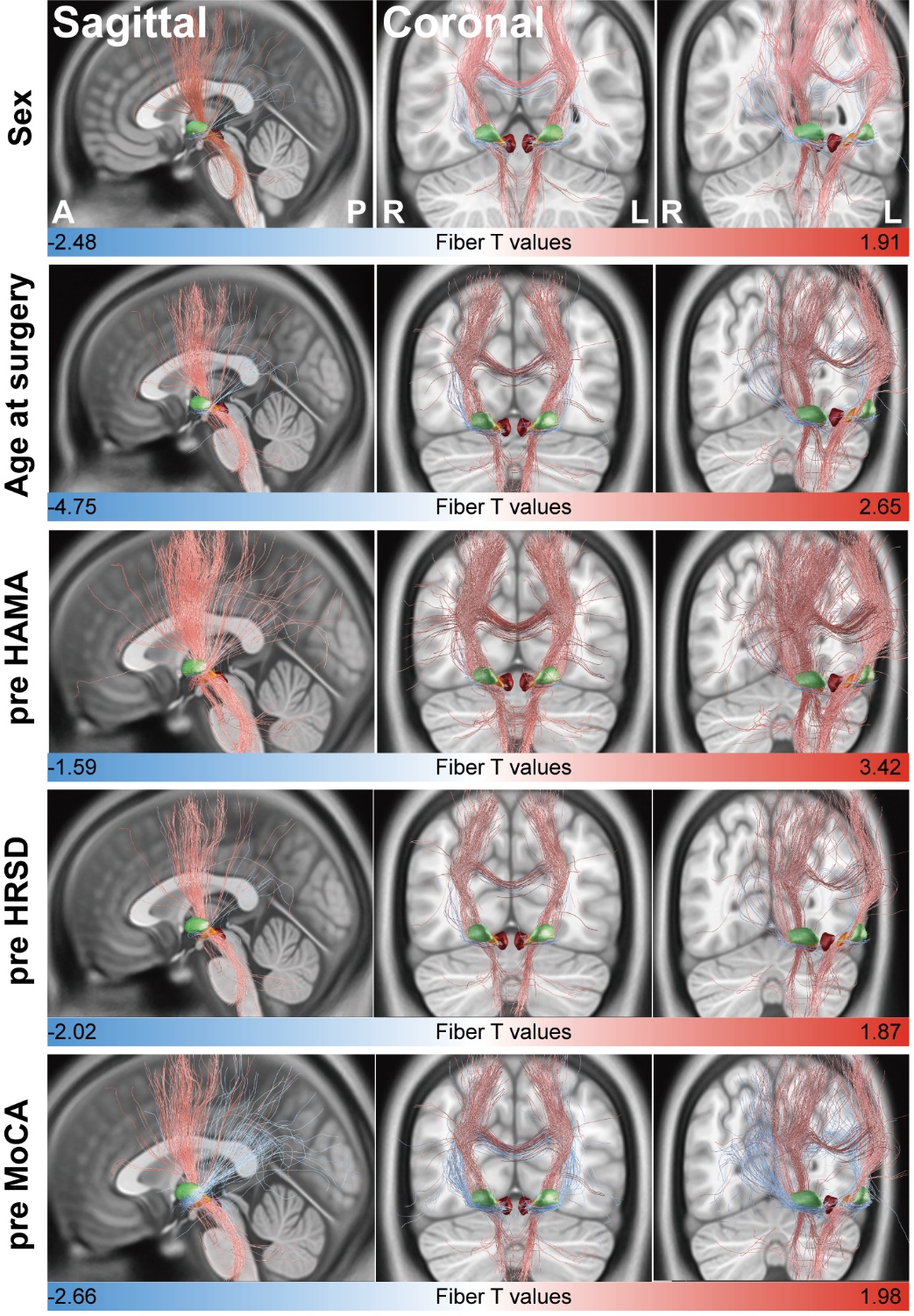


**Supplemental Figure 8:** Reconstruction of fiber tracts associated with optimal improvement of TWSTRS severity score in (A) GPi-DBS, (B) STN-DBS and (C) the whole dataset.


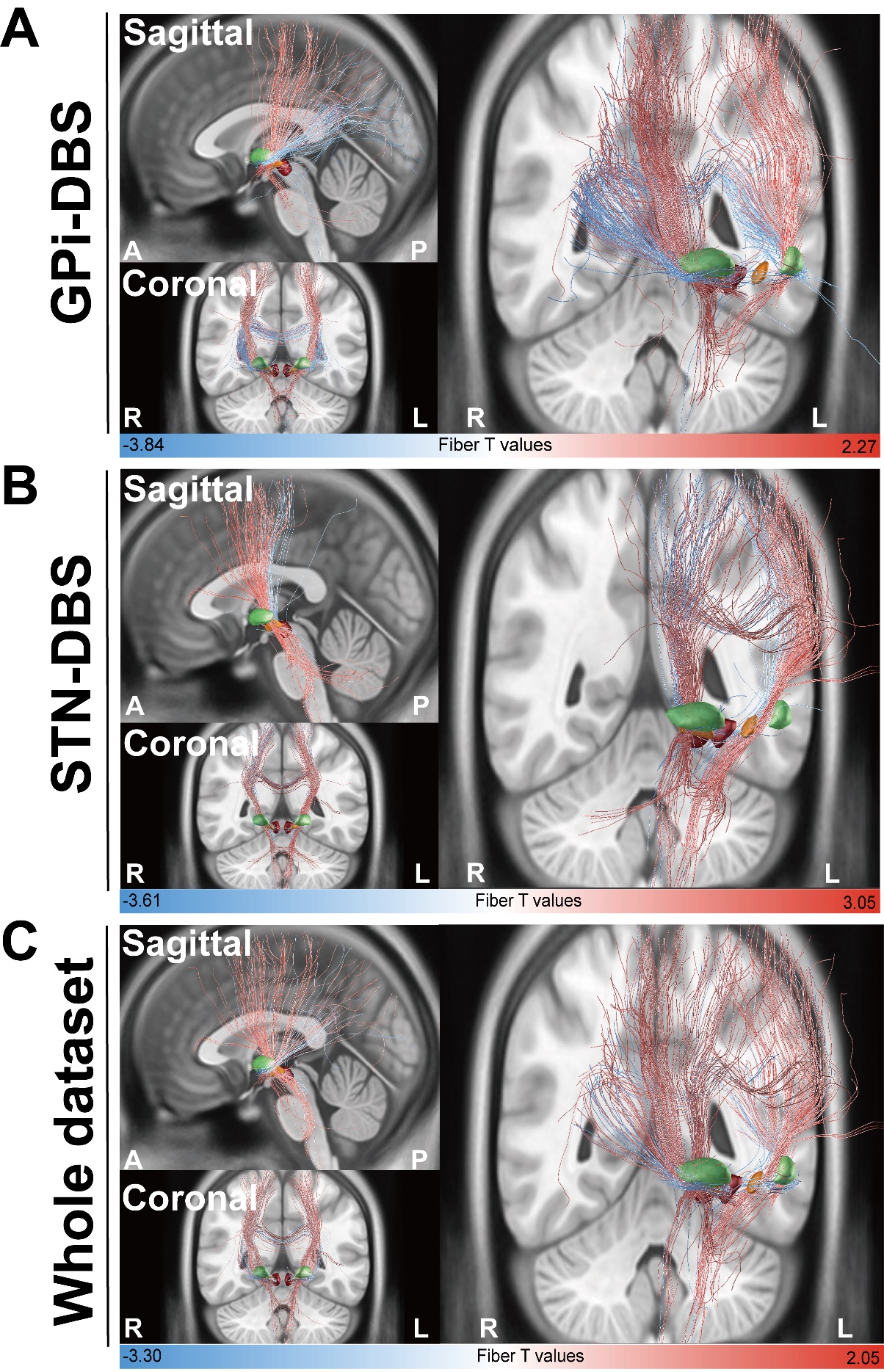


**Supplemental Figure 9:** Functional connectivity profile of cohorts associated with the improvement of TWSTRS total score divided by target regions after adjusting covariates (such as sex, age at surgery, pre HAMA scores, pre HRSD scores, and pre MoCA scores).


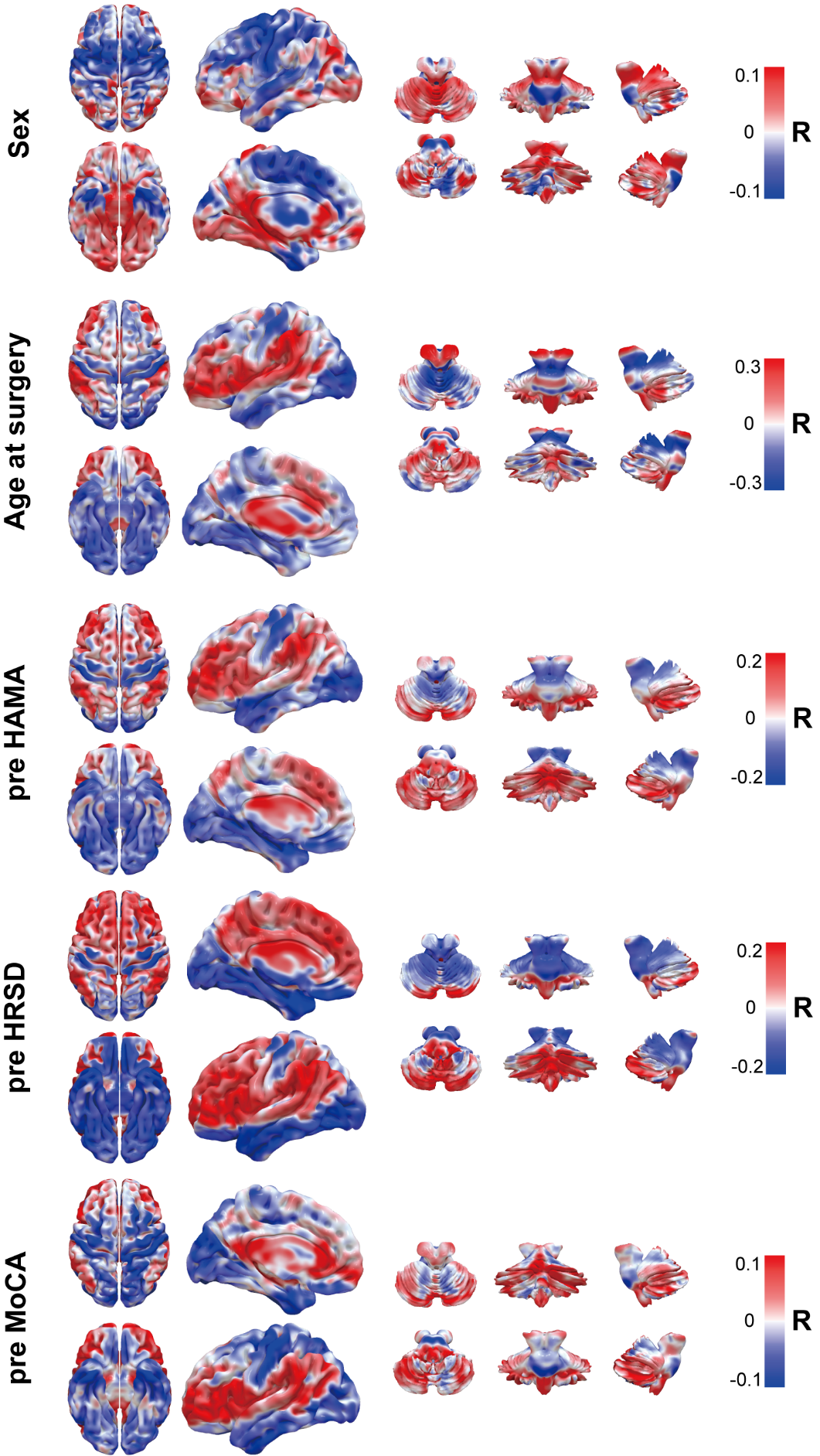


**Supplemental Figure 10:** Functional connectivity profile of cohorts associated with the improvement of TWSTRS severity score divided by target regions from (A) GPi-DBS, (B) STN-DBS, (C) the whole dataset and (D) agreement map based on the GPi and STN-DBS.


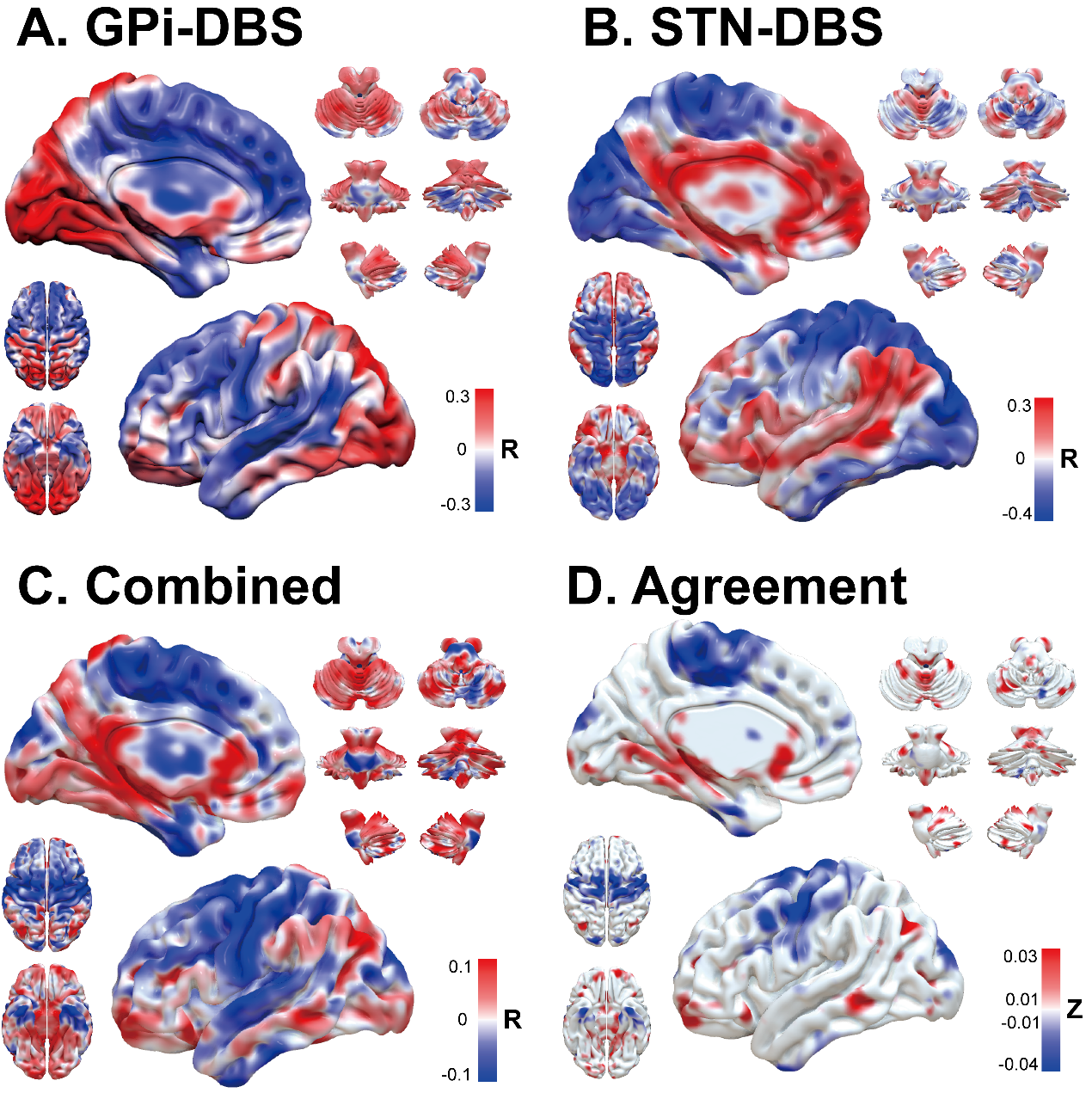


**Supplemental Table 1: Inclusion and exclusion criteria**

| **Inclusion criteria:** |
| --- |
| 1. ages 18-75; 2. meet the diagnostic criteria for cervical dystonia 3. receive bilateral GPi- or STN-DBS treatment 4. the optimal parameters are achieved within 6 months after surgery 5. failed treatment with oral medications and/or botulinum toxin injections 6. medical history ≥ 1 year 7. normal cognitive function, MoCA≥24; 8. the subject or his legal representative can sign the informed consent. |
| **Exclusion criteria:** |
| 1. dystonia elsewhere in the body 2. dystonia caused by other secondary factors (including birth injury, traumatic brain injury and psychiatric treatment, etc.) 3. with other neuropsychiatric diseases (Alzheimer's disease, amyotrophic lateral sclerosis, Parkinson's disease, etc.) 4. patients with previous history of other surgery for CD such as SPD 5. major depressive disorder (HRSD>35) 6. missing data or lost follow-up |

**Supplemental Table 2: Univariate regression analysis of improvement rate of TWSTRS total score with preoperative clinical and demographical features.**

| Variables | Univariate regression | | |
| --- | --- | --- | --- |
|  | Slope | 95% confidence interval | *p* |
| Stimulation target | 0.0004621 | -0.007282 to 0.008206 | 0.9028 |
| Sex | -0.0003353 | -0.008130 to 0.007460 | 0.9299 |
| Age at surgery | -0.2548 | -0.4032 to -0.1064 | **0.0017** |
| Follow-up | 0.1094 | -0.1388 to 0.3575 | 0.3713 |
| TWSTRS total | -0.1312 | -0.2996 to 0.03729 | 0.1209 |
| TWSTRS severity | -0.03916 | -0.1032 to 0.02484 | 0.2183 |
| TWSTRS disability | -0.07869 | -0.1631 to 0.005675 | 0.0661 |
| TWSTRS pain | -0.01027 | -0.09749 to 0.07695 | 0.8097 |
| HAMA | -0.07670 | -0.1213 to -0.03214 | **0.0017** |
| HRSD | -0.08038 | -0.1485 to -0.01225 | **0.0228** |
| MoCA | 0.02874 | 0.009449 to 0.04804 | **0.0053** |

TWSTRS: Toronto Western Spasmodic Torticollis Rating Scale; HAMA: Hamilton anxiety scale; MoCA: Montreal cognitive assessment; HRSD: Hamilton rating scale for depression; VIF: variance inflation factor.

Bold indicates statistical significance.

**Supplemental Table 3: Stereotactic coordinates of the active contacts at follow-up settings in Montreal Neurological Institute (MNI) space (mm).**

| Training group | Right-side hemisphere | | | Left-side hemisphere | | |
| --- | --- | --- | --- | --- | --- | --- |
| ID | **X** | **Y** | **Z** | **X** | **Y** | **Z** |
| GPi-DBS | | | | | | |
| #01 | 23.36 | -4.14 | -2.41 | -21.83 | -4.22 | -7.37 |
| #02 | 24.55 | -6.05 | -7.31 | -22.37 | -7.50 | -8.89 |
| #03 | 23.50 | -5.26 | -3.05 | -20.13 | -9.67 | -8.13 |
| #04 | 23.05 | -7.62 | -5.84 | -22.88 | -8.91 | -6.95 |
| #05 | 21.77 | -6.95 | -8.10 | -21.51 | -9.15 | -5.89 |
| #06 | 22.11 | -6.50 | -3.65 | -22.62 | -7.19 | -3.75 |
| #07 | 21.62 | -9.38 | -9.27 | -21.71 | -11.11 | -10.49 |
| #08 | 23.14 | -6.03 | -5.77 | -21.05 | -8.22 | -7.92 |
| #09 | 23.97 | -9.18 | -5.79 | -23.14 | -8.87 | -5.94 |
| #10 | 22.33 | -4.60 | -4.37 | -20.75 | -5.95 | -3.88 |
| #11 | 23.11 | -11.13 | -10.36 | -20.27 | -8.62 | -7.51 |
| #12 | 20.91 | -9.55 | -10.62 | -20.46 | -8.00 | -9.07 |
| #13 | 22.06 | -7.59 | -6.79 | -19.98 | -8.51 | -7.53 |
| #14 | 18.92 | -8.92 | -7.89 | -21.56 | -8.56 | -7.44 |
| STN-DBS | | | | | | |
| #15 | 13.20 | -16.08 | -10.84 | -10.37 | -16.13 | -9.89 |
| #16 | 14.43 | -10.59 | -5.63 | -13.43 | -10.76 | -5.54 |
| #17 | 12.61 | -14.63 | -8.97 | -10.83 | -14.27 | -7.98 |
| #18 | 14.29 | -12.94 | -6.32 | -13.16 | -13.06 | -9.12 |
| #19 | 14.48 | -15.46 | -8.16 | -12.64 | -16.10 | -7.64 |
| #20 | 14.73 | -13.30 | -7.61 | -11.51 | -13.23 | -6.94 |
| #21 | 15.64 | -12.51 | -6.35 | -11.85 | -11.84 | -4.79 |
| #22 | 12.66 | -12.51 | -7.84 | -11.53 | -12.51 | -8.53 |
| #23 | 13.41 | -9.79 | -3.09 | -13.29 | -11.47 | -5.23 |
| #24 | 14.72 | -13.97 | -6.48 | -13.41 | -13.01 | -5.44 |
| #25 | 11.65 | -15.53 | -10.27 | -10.48 | -15.75 | -9.16 |
| Test group | Right-side hemisphere | | | Left-side hemisphere | | |
| ID | **X** | **Y** | **Z** | **X** | **Y** | **Z** |
| GPi-DBS | | | | | | |
| #26 | 23.19 | -6.7 | -2.02 | -20.26 | -7.34 | -5.35 |
| #27 | 22.61 | -6.78 | -4.47 | -21.37 | -6.25 | -4.51 |
| #32 | 24.02 | -6.23 | -7.95 | -21.04 | -8.2 | -8.89 |
| #33 | 20.29 | -6.8 | -5.7 | -23.36 | -9.2 | -2.11 |
| #34 | 24.03 | -7.89 | -3.85 | -21.92 | -9.67 | -4.09 |
| #35 | 20.95 | -7.71 | -8.46 | -24.28 | -7.61 | -4.85 |
| #36 | 20.03 | -5.66 | -4.19 | -20.45 | -9.25 | -6.72 |
| #37 | 22.57 | -7.11 | -9.5 | -22.04 | -9.08 | -5.01 |
| STN-DBS | | | | | | |
| #28 | 12.32 | -10.92 | -7.61 | 13.51 | -12.02 | -8.77 |
| #29 | 11.11 | -15.49 | -4.62 | 11.11 | -9.78 | -3.48 |
| #30 | 12.16 | -10.37 | -9.09 | 12.74 | -14.48 | -9.78 |
| #31 | 11.87 | -11.78 | -6.3 | 11.38 | -9.16 | -7.78 |
| #38 | 13.87 | -10.23 | -9.87 | 13.08 | -9.16 | -2.44 |

X coordinate: medial-lateral direction (lateral); Y coordinate: anterior-posterior direction (axial); Z coordinate: dorsal-ventral direction (vertical).
